# Research on prediction algorithm of effluent quality and development of integrated control system for waste-water treatment

**DOI:** 10.1038/s41598-025-03612-5

**Published:** 2025-06-02

**Authors:** JianWun Lai

**Affiliations:** School of Engineering, University of California San Diego, San Diego, CA 92093 USA

**Keywords:** Waste-water treatment, Integrated control system, CNN, LSTM, Energy consumption, Chemical oxygen demand, Accuracy, Environmental sciences, Environmental social sciences

## Abstract

Research is implemented to protect the environment from an epidemic of chemical materials that could render living conditions hazardous. In order to efficiently use productivity while maintaining a constant and reliable level of waste quality, severe regulations regarding Waste-Water Treatment and Control Systems (WWTCS) must be adopted to mitigate the serious nature of water pollution and impure performance. Suboptimal treatment efficiency and use of resources are the results of the methods used for WWTCS, which are not highly susceptible to changing impact features and complex biological systems. The present study presented a prediction algorithm and an Integrated Control System (ICS) to address the problems of conventional methods. This research proposes a Deep Learning (DL) for the quality of wastewater prediction that employs a Quantile Regression-Random Forest (QR-RF) meta-learner when combined with Convolutional Neural Networks (CNN), Long Short-Term Memory (LSTM), and Gated Recurrent Units (GRU). The proposed method has been implemented into practice and tested at Asia’s Jiangsu Province Metropolitan Waste-Water Treatment Plant (WWTP). With a Root Mean Absolute Error (RMSE) of 4.76 mg/L for 24-h horizons and a Mean Absolute Error (MAE) of 0.85 mg/L for 1-h predictions, the proposed model outperforms conventional methods in terms of prediction accuracy. The ICS is superior to standard WWTCS by a vital error boundary, minimizing energy consumption by 17% and boosting chemical-based consumption optimization by 24%. With an average removal rate of 94.23% for Chemical Oxygen Demand (COD) compared to 88.76% for standard systems, the findings from experiments exhibited significant performance improvements.

## Introduction

Waste-water treatment plants (WWTP) play a critical role in restoring public health and preventing pollution by processing vast volumes of wastewater from industrial and domestic sources^1,2^. In order to achieve maximum operational efficiency while maintaining consistent wastewater quality, Waste-Water Treatment and Control Systems (WWTCS) involve challenging chemical and biological processes^3,4^. However, due to their emphasis on human intervention and static operational variables, standard WWTP maintenance and control methods struggle to adapt to ever-changing influent features and variable environmental factors^5^. This situation and the need to meet stringent regulatory standards and control operational expenses present key challenges in managing modern WWTPs^1,6^. According to the World Health Organisation Ecological Programme (WHOEP), approximately 80% of global wastewater still flows untreated, highlighting the urgent necessity for improved treatment technologies^7^. Furthermore, industrial development has led to more complex wastewater compositions, requiring enhanced treatment processes^8^, while the high energy usage of WWTPs, contributing approximately 25–40% of total public energy costs, emphasizes the need for adaptive control methods^9^. Despite developments, significant knowledge gaps remain in the real-time optimization and predictive management of WWTCS.

To address these operational complexities, Artificial Intelligence (AI) technologies and sensor devices have emerged as promising solutions for managing WWTP processes. Deep Learning (DL) models have demonstrated the capacity to manage the non-linear and time-dependent relationships inherent in WWTCS, enabling improvements in time-series prediction and process control. Several methods have been proposed: Artificial Neural Networks (ANN) and shallow models have been applied for wastewater quality prediction^10^ but typically suffer from limited adaptability and lack of dynamic control integration. IoT models^11^ have improved real-time monitoring but frequently fall short in predictive analytics and control optimization. DL, such as hybrid CNN + GRU^12^ and LSTM^13^, have advanced effluent quality prediction by capturing complex spatial and temporal patterns, yet existing implementations primarily focus on static predictions without seamless integration into operational control systems. Physics-informed and transfer learning models^14^ have attempted to address data scarcity but are controlled in real-time adaptability. Multimodal methods combining imaging and water quality data^15^ present additional perceptions but high computational complexity, limiting real-time deployment feasibility. Economic model predictive control using learning-based Koopman operators^16^ enhances operational efficiency but lacks direct effluent quality prediction focus. Moreover, Model-Free Reinforcement Learning (MFLR)^17^ propose adaptive control approaches but faces challenges regarding interpretability and industrial readiness. Additionally, Feed-Forward Neural Networks (FFNN) combined with optimization methods^18^ have demonstrated limited generalization in dynamic operational contexts. Consequently, a critical gap remains in developing a predictive, uncertainty-aware, and operationally integrated control solution for WWTPs.

Despite the growing body of research employing DL for effluent prediction, existing methods display significant drawbacks. Conventional models such as LSTM, CNN-Attention hybrids, or physics-informed models frequently fail to integrate uncertainty quantification and real-time operational decision support in a unified framework. Moreover, few studies incorporate predictive analytics tightly into Integrated Control Systems (ICS) to optimize treatment performance and resource consumption dynamically. The novelty of the proposed methodology lies in (i) designing a practical ensemble DL that captures spatial and temporal wastewater dynamics, (ii) integrating a Quantile Regression-Random Forest (QR-RF) meta-learner for robust uncertainty-aware predictions, and (iii) implementing an operationally deployable ICS platform (AQUAtec) capable of real-time monitoring, control, and decision support. This method uniquely bridges predictive modelling and operational control, addressing methodological and practical gaps in existing WWTCS solutions.

The objectives and main contributions of this study are summarized as follows:

- Implementation of an ensemble DL (CNN + LSTM + GRU) for predicting wastewater quality across diverse operational environments.
- Development of an ICS to apply predictive analytics for real-time workflow optimization.
- Validation of the system’s performance using two years of operational data from a large-scale municipal WWTP.
- Detailed assessment of system adaptability and performance under numerous operational scenarios.
- Integration of uncertainty-aware predictions into operational decision support to enhance reliability and control efficiency.

This paper reviews recent developments in WWTCS, DL applications in process prediction, and integrated automation tactics. It details the methodology, including the network model, Prediction Algorithm (PA), and ICS implementation. The experimental setup at Jiangsu Municipal WWTCS, data collection procedures, and implementation protocols are described. Results and analysis compare the proposed model with existing approaches across multiple operational factors. This study concludes with key results, practical implications, and directions for future research in WWTCS automation.

## Literature review

ANN has been used to predict the performance of WWTCS, with a non-linear transformation model demonstrating improved accuracy in predicting waste quality based on multiple pollution variables^10^. The combination of IoT and AI has attempted to overcome the challenges faced by traditional WTCS. This combination enables real-time monitoring and predictive maintenance, enhancing the system’s responsiveness and operational efficiency^11^.

Optimized DL, such as CNN, are combined with GRU^12^ to predict effluent COD and TSS. These hybrid models have shown better generalization ability and achieved a high coefficient of determination (R^2^) for COD and TSS predictions.^13^ had applied the LSTM within Benchmark Simulation Model No. 2 (BSM2) to enhance the predictability and efficiency of WTCS.^14^ had tried to improve the dissolved oxygen concentration predictions in industrial WWTPs by combining Transfer Learning (TL) with physics-informed modelling. Their model employed data from simulation models and other industrial plants to enhance prediction performance and tried to address the challenges related to data quality and availability.

Authors^15^ have presented a prediction model for effluent quality based on neural processing; they trained their model on integrated multimodal data that includes key parameters and waste-water surface images. They experimented and validated their model against the model for prediction accuracy for effluent COD and ammonia levels.

An economic model predictive control-based model applying learning-based Koopman operators^16^ to optimize WTCS. Their model focused on improving economic operational performance by managing Energy Consumption (EC) and treatment costs. The development of ICS based on AI has been emphasized to manage the complexities of WWTP operations^17^. These systems aim to ensure effluent quality that aligns with environmental regulations while minimizing operating costs through.

An enhanced FFNN was developed by^18^ to predict WWTP effluent quality. They developed optimization algorithms to enhance FFNN’s real-time WWTP effluent quality prediction efficiency. They applied their model to predict effluent COD and total nitrogen levels using hourly water quality parameters and operational data. Advancements in Sequencing Batch Reactors (SBR) have been reviewed in^19^, and they highlighted its operational flexibility and process control capabilities. SBRs are extensively used for treating WWTCS contaminated with complex pollutants, with various configurations and operational modifications developed to meet upgraded effluent limits.

Recently, significant progress has been made in integrating Machine Learning (ML) and DL for water quality monitoring and classification tasks.^20^ suggested an approach combining Feature Extraction (FE) such as Principal Component Analysis (PCA), Linear Discriminant Analysis (LDA), and Independent Component Analysis (ICA) with Long Short-Term Memory-Recurrent Neural Networks (LSTM-RNN) to improve the classification accuracy for drinking water quality, achieving an impressive accuracy of 99.72% on datasets from the Tilesdit Dam in Algeria. Their study emphasized the potential of DL, particularly LSTM, in handling large-scale smart monitoring data. Similarly,^21^ presented a decision fusion framework based on combining Support Vector Machines (SVMs) and ANN using decision templates, enhanced with PCA-based Feature Selection (FS), generating a classification accuracy of 99.24% for surface water quality assessment.

A detailed review of existing studies, summarized in Table 1, reveals several persistent gaps in predictive analytics and control of wastewater treatment. Prior works have predominantly focused on improving prediction accuracy for specific parameters (*e.g.,* COD, DO, TSS) but often lack integration into operational control models. Moreover, most methods neglect uncertainty quantification, rely heavily on simulation environments without industrial-scale validation, or present complexity barriers for real-time deployment. Furthermore, decision-support aspects that translate predictive insights into actionable control strategies are seldom addressed. The proposed model uniquely addresses these limitations by combining a robust ensemble DL (CNN + LSTM + GRU) with Quantile Regression-Random Forest (QR-RF) uncertainty quantification and integrating the prediction pipeline directly into a real-time operational ICS, thereby enabling accurate, reliable, and actionable wastewater treatment optimization.

**Table 1:** Limitations in the literature.

References	Method used	Focus	Limitation	Gap addressed by this work
^10^	ANN	Wastewater Quality Prediction	Limited to shallow models; lacks uncertainty modelling	Introduce deep ensemble with uncertainty quantification
^11^	IoT + AI for WWTP	Real-time Monitoring and Predictive Maintenance	Focused on monitoring; no real-time control integration	Integrate predictive control with ICS
^12^	Hybrid CNN + GRU	COD, TSS Prediction	Focused on accuracy; lacks uncertainty bounds and control system integration	Add robust uncertainty prediction and ICS coupling
^13^	LSTM on BSM2 Data	Effluent Prediction	Simulation-focused; limited industrial validation	Validate on large-scale real WWTP data
^14^	TL + Physics models	Dissolved Oxygen prediction	Focused on specific parameters; lacks general wastewater quality modelling	Broaden multi-parameter effluent quality prediction
^15^	Multimodal (Images + Water Data)	Effluent COD and Ammonia Prediction	High complexity; difficult for real-time ICS deployment	Provide operationally deployable solution
^16^	Learning-based Koopman Operator	Economic Model Predictive Control	Focused on economic optimization, not quality prediction	Integrate quality prediction with operational control
^17^	Model-Free RL Control	Intelligent Control	Complex training; lacks interpretability	Use interpretable ensemble models
^18^	FFNN + optimization	Effluent quality prediction	Shallow model; limited generalization	Deploy robust ensemble DL

## Methodology

**Fig. 1:**
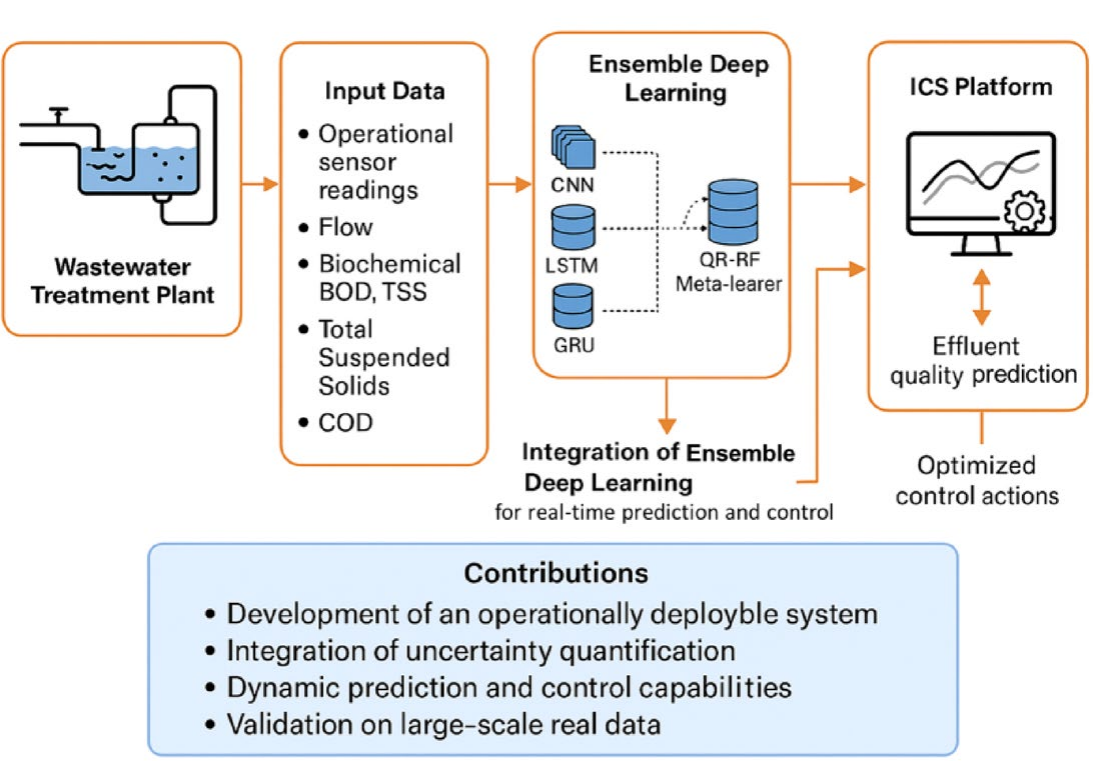
Global schematic of the proposed integrated control and prediction model for wastewater treatment optimization.

This section presents the proposed integrated model for wastewater treatment prediction and control. The system is designed to leverage IoT-enabled sensor networks, ensemble DL, and real-time ICS operations to optimize effluent quality and operational efficiency. The overall design of the proposed model is illustrated in Fig. 1.

The global schematic presented in Fig. 1 illustrates the complete workflow of the proposed approach, beginning with IoT-based sensor data acquisition and proceeding using advanced predictive modelling and real-time control integration. Unlike existing methodologies focusing solely on effluent prediction or isolated control improvements, the proposed model uniquely combines ensemble DL with quantile-based uncertainty quantification and operational deployment through an ICS platform. This integrated architecture enables accurate effluent quality prediction and actionable decision support for optimizing chemical usage, energy efficiency, and treatment performance, thus addressing methodological and practical challenges in modern wastewater treatment operations.

### Proposed IoT model for WWTP

**Fig. 2:**
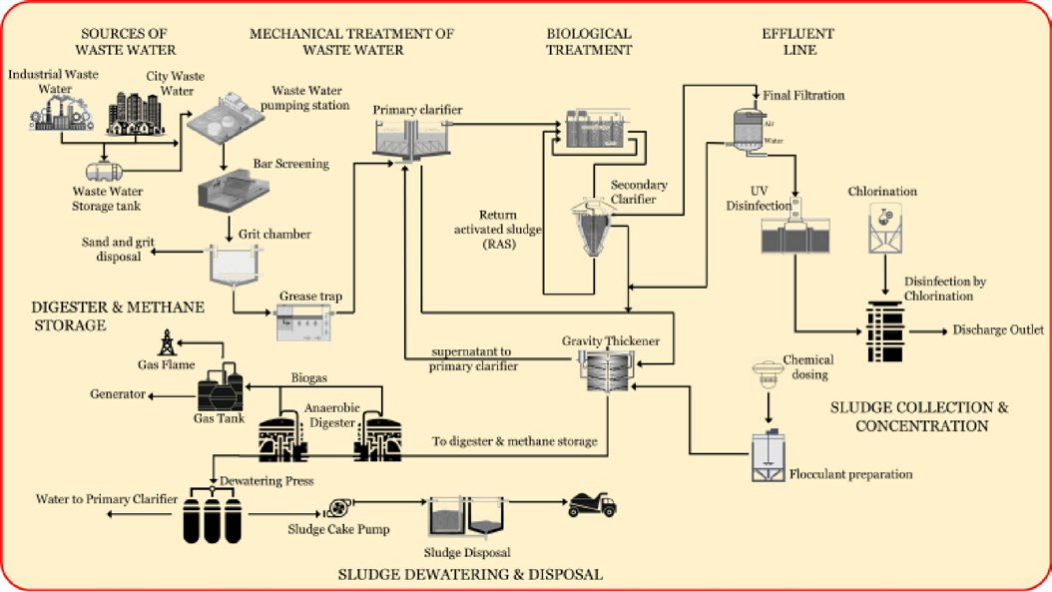
Model of WWTP.

The general model of the WWTP (Fig. 2) integrates several physical, chemical, and biological treatment stages. The proposed IoT for the WWTP integrates IoT-enabled sensors, Edge Computing, a centralized data management system, and a monitoring and management interface. The model comprises three primary layers: IoT sensor deployment, data processing and storage, and the monitoring and management interface.

**Fig. 3:**
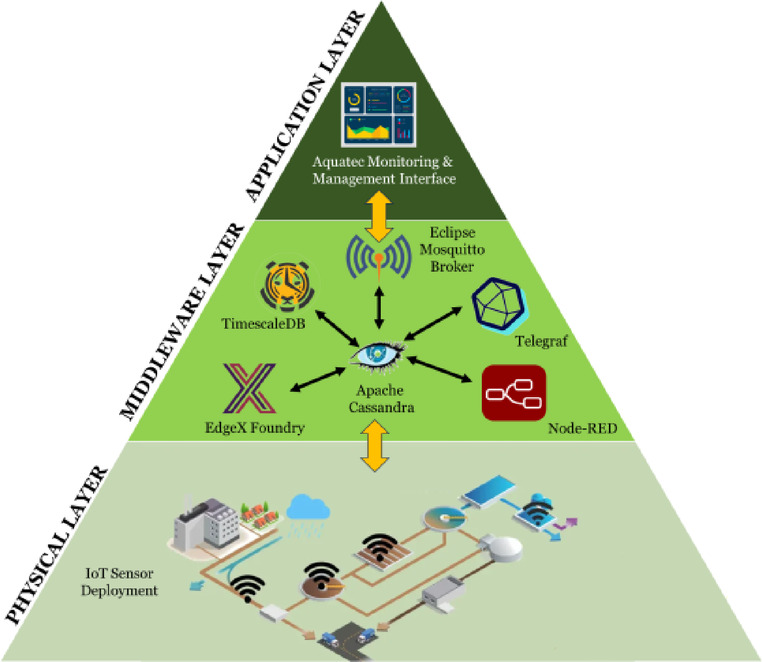
IoT Model.

In the proposed model, the IoT is pivotal as the preliminary layer for real-time, high-fidelity data acquisition, mode responsiveness, and operational feedback (Fig. 3). The system continuously monitors key parameters such as flow rates, liquid levels, pH, dissolved oxygen, and chemical concentrations by deploying a dense network of IoT-enabled sensors across critical stages of the wastewater treatment process. This granular and timely data collection forms the vital input for the predictive analytics module, enabling accurate effluent quality prediction. Moreover, IoT devices enable decentralized intelligence through local data preprocessing (edge computing), reducing latency, network load, and system response times. IoT ensures resilient, scalable, and secure data transmission to centralised processing systems through MQTT-based communication protocols and robust data persistence mechanisms (e.g., Apache Cassandra, TimescaleDB). Without the IoT setup, the real-time dynamic sensing and responsive control necessary for optimizing chemical dosing, energy consumption, and effluent quality would not be feasible. Thus, IoT is not merely a monitoring tool but also a critical enabler of predictive control and operational optimization in the proposed WWTP model.

(i) IoT sensor deployment layer
  The model begins with the deployment of IoT-enabled sensors across the WWTP. These sensors are installed at key locations, such as waste-water storage tanks, grit chambers, primary and secondary clarifiers, biological treatment units, and sludge management facilities. They continuously monitor parameters, including pH, turbidity, dissolved oxygen, temperature, flow rate, and sludge levels. The sensors are equipped with wireless communication modules, enabling them to transmit data in real-time to the next layer of the model.
(ii) Data processing and storage layer
  This layer is responsible for collecting, processing, and storing the data generated by the IoT sensors.
  The components in this layer include:
  EdgeX Foundry: The computing model processes raw sensor data locally to reduce latency and bandwidth usage.
  Apache Cassandra: Sensor data is stored in Cassandra for historical data analysis and ML applications.
  TimescaleDB: It enables storing and retrieving historical trends for predictive analytics and system optimization.
  Telegraf: An open-source agent responsible for collecting, processing, and transmitting data to the database.
  Eclipse Mosquitto Broker: A lightweight Message Queue Telemetry Transport (MQTT) protocol-based message agent that helps communication between IoT devices and the central system.
  Node-RED: A flow-based development tool used to design workflows for data processing and control actions.
(iii) Monitoring and management interface
  The top layer of the model is the Aquatec Monitoring and Management Interface, which serves as a centralized dashboard for operators to visualize, analyze, and control the processes within the WWTP. This interface provides real-time monitoring capabilities, displaying live data from sensors, such as flow rates, pH levels, and dissolved oxygen, on a graphical interface that allows operators to identify and address problems as they arise quickly.

#### Sensor deployment layer

**Fig. 4:**
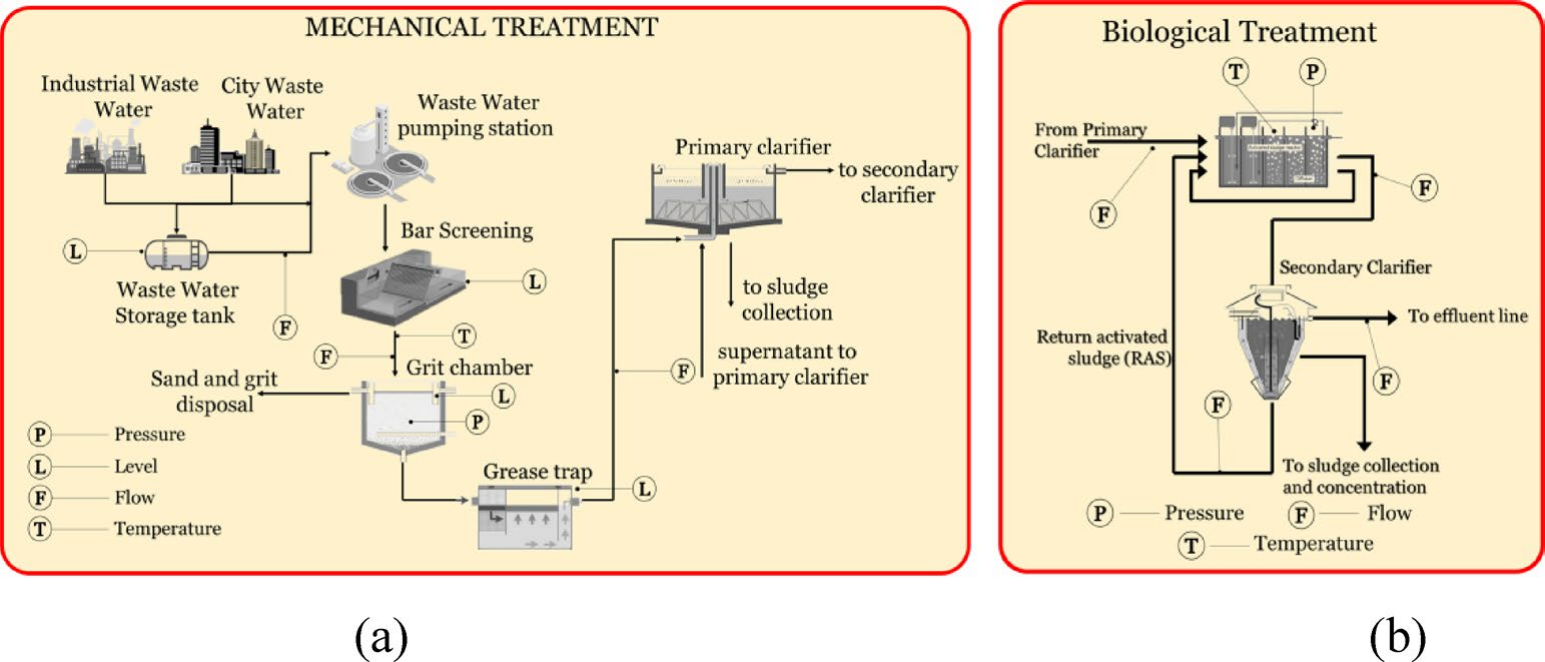
Sensors deployed at (**a**) mechanical treatment and (**b**) biological treatment.

The operation of the WWTP relies on a complete network of sensors deployed across critical points in the treatment process. In the mechanical treatment phase (Fig. 4a), the sensor is deployed at the source points, monitoring industrial and city wastewater inputs. Level sensors in the waste-water storage tank maintain storage conditions, and flow sensors regulate the intake rates of the pumping station. The bar screening and grit chamber units have level and temperature sensors to monitor debris accumulation and process conditions.

The biological treatment stage (Fig. 4b) combines temperature and flow sensors. The temperature sensors help maintain favourable conditions for biological processes, while flow sensors Regulate Sludge Recirculation (RAS) and effluent outflow to the clarifiers.

**Fig. 5:**
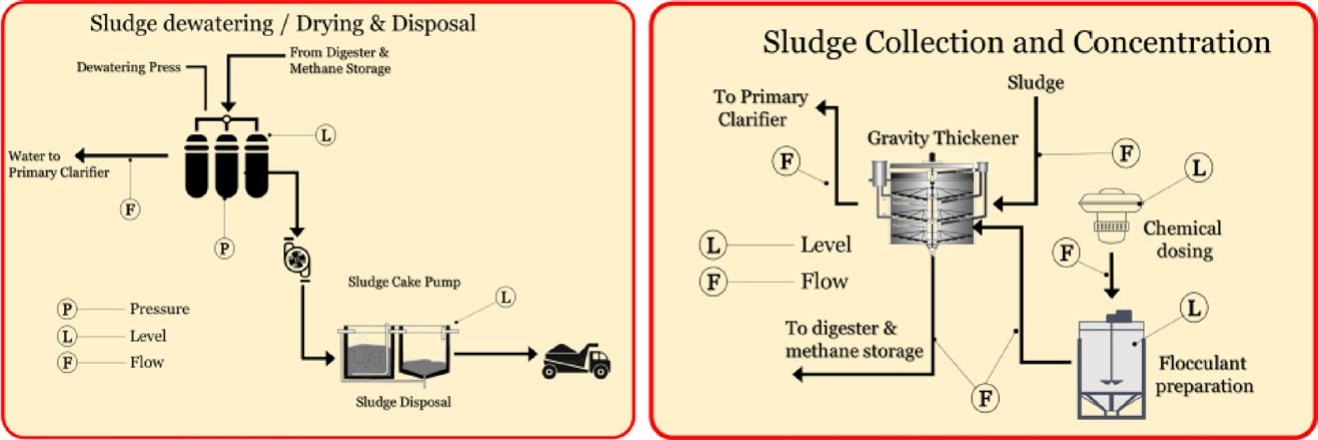
Sensors deployed at (**a**) sludge collection and concentration unit; (**b**) sludge dewatering and disposal stage.

In the sludge collection and concentration unit (Fig. 5a), level sensors are installed to monitor sludge accumulation in the gravity thickener, while flow sensors track the movement of sludge and chemical dosing agents. The sludge dewatering and disposal stage (Fig. 5b) employs pressure sensors to monitor the operation of dewatering presses and flow sensors to regulate the transfer of sludge into drying units or disposal systems.

**Fig. 6:**
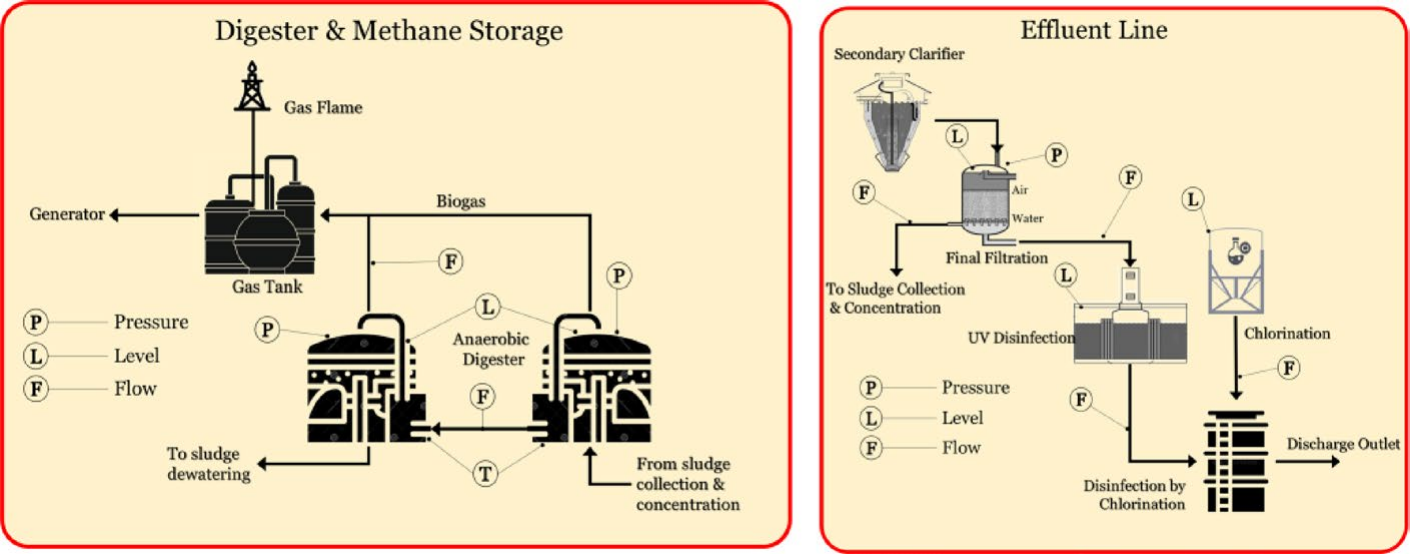
Sensors deployed at (**a**) digester and methane storage unit; (**b**) effluent line.

Level sensors in the digester and methane storage unit (Fig. 6a) monitor sludge input, while temperature sensors ensure that the digester operates within the optimal temperature range for microbial activity. Flow sensors measure the movement of sludge and biogas, while pressure sensors monitor gas storage tanks to ensure safe methane handling. The effluent line (Fig. 6b) uses pressure, level, and flow sensors. The flow sensors measure the final discharge rates to track compliance and operational efficiency.

All sensors in the network are interconnected through a wireless mesh topology, ensuring reliable data transmission even in challenging industrial environments. The sensor network uses industrial-grade IoT devices with IP67 security ratings, capable of enduring the corrosive and humid conditions typical in WWTC facilities. Each sensor node incorporates local processing capabilities for preliminary data filtering and validation, reducing network load and enabling rapid response to critical conditions.

#### Middleware layer

**Fig. 7:**
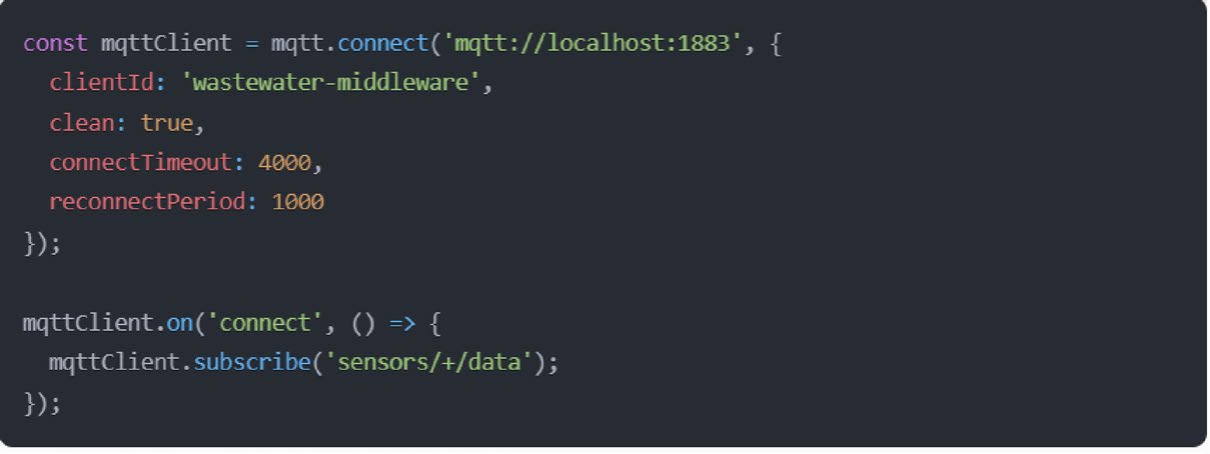
MQTT agent configuration and subscription management.

The middleware layer is the critical intermediary infrastructure in the proposed WWTC system, orchestrating data flows between physical IoT sensors and the application layer. Let me articulate the key components and their implementation, supported by relevant code examples. At the core of the proposed middleware layer is the Message Agent System (MSM), which is implemented using Eclipse Mosquitto and handles MQTT protocol communications. The agent configuration demonstrates robust error handling and automatic reconnection capabilities (Fig. 7).

**Fig. 8:**
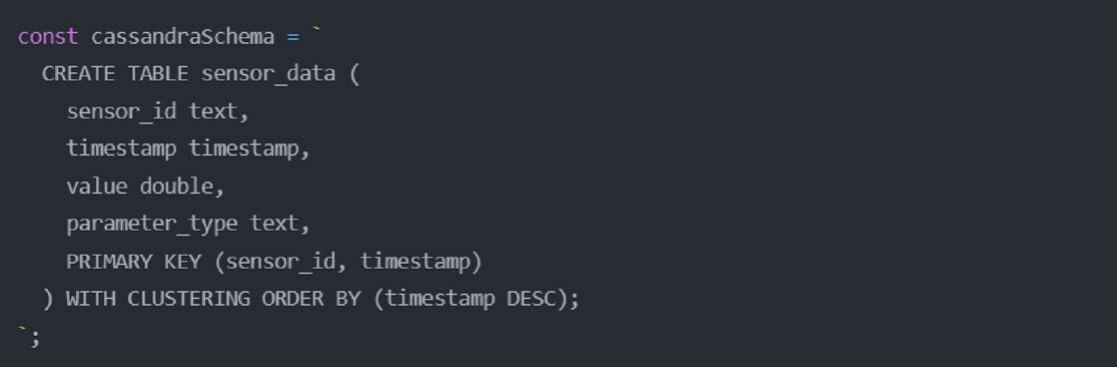
Cassandra database schema definition.

Data persistence is managed using a dual-database approach. Apache Cassandra is our primary data store, handling high-throughput sensor readings with a schema optimized for WWTCS parameters (Fig. 8).

**Fig. 9:**
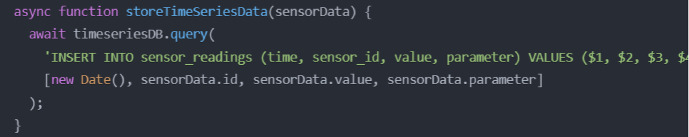
TimescaleDB time-series data management.

For specialized time-series analytics, this study deploys TimescaleDB, which provides efficient storage and querying of temporal data patterns. The implementation includes automated data retention policies and aggregation functions (Fig. 9).

**Fig. 10:**
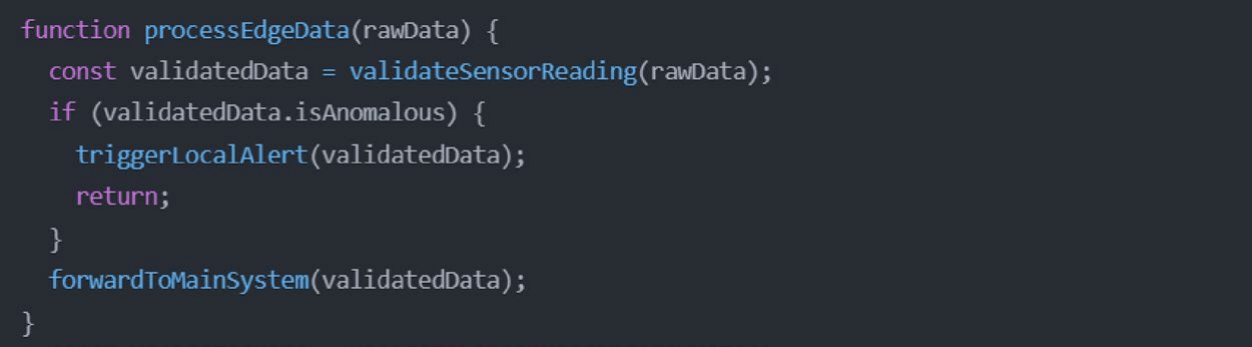
EdgeX foundry edge computing implementation.

The data processing pipeline incorporates edge computing capabilities through EdgeX Foundry, enabling preliminary data validation and filtering at the network edge. This reduces central system load and enables rapid response to critical conditions (Fig. 10).

**Fig. 11:**
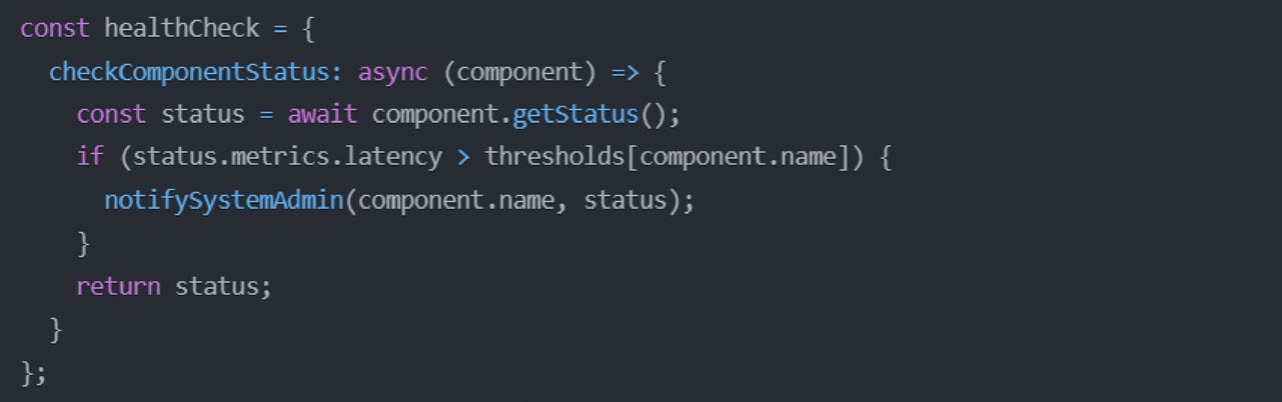
System health monitoring service.

System health monitoring is implemented through a comprehensive checking mechanism that tracks component status and performance metrics (Fig. 11).

#### Predictive analytics using ensemble CNN + LSTM + GRU with QR-RF in the application layer

The predictive modelling framework for this study was designed around DL, particularly CNN models like (CNN, CNN + LSTM, CNN + GRU), due to their proven ability to model complex spatial–temporal dependencies inherent in wastewater treatment system data. CNNs are highly effective at extracting localized spatial patterns from multivariate time-series sensor data, enabling the model to learn correlations between features such as flow, pH, DO, COD, and TSS over short-term intervals. However, wastewater systems also exhibit strong temporal dependencies across more extended periods (*e.g.,* sludge settling behaviour or delayed chemical effects), which motivated the integration of sequence modelling layers such as LSTM + GRU after the CNN-based FE. LSTM layers are particularly suited for capturing long-term temporal dependencies due to their gated memory structure, while GRUs present a computationally lighter alternative with competitive performance.

**Fig. 12:**
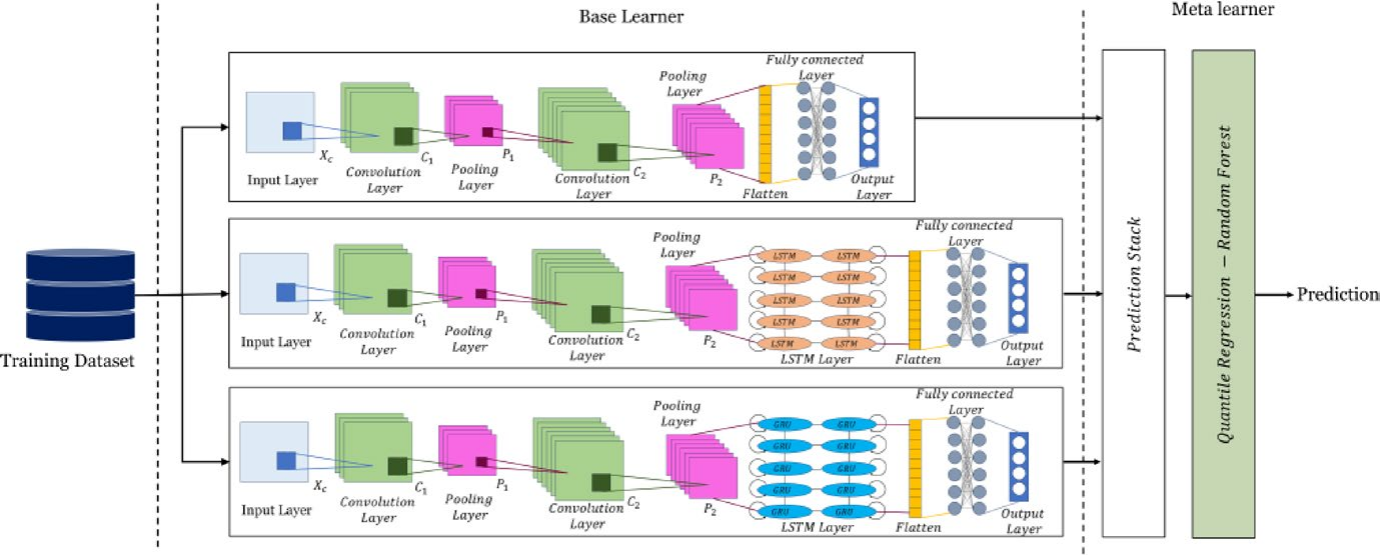
Predictive analytics using ensemble CNN + LSTM + GRU with QR-RF.

The predictive analytics model (Fig. 12) for effluent quality prediction integrates an ensemble of DL and QR-RF. The input to the predictive model consists of sensor data collected from numerous stages of the WWTCS. This data is denoted as ${\mathbf{X}}_{c}$, is a time-series matrix with dimensions $T\times$ $F$, where $T$ represents the time steps and $F$ the number of features. Each base learner processes this data independently for relevant FE and makes predictions. The QR-RF meta-learner then aggregates these predictions to generate the final output, denoted as ${\mathbf{\hat{Y}}}$, which includes point predictions and uncertainty bounds.

The first base learner in the ensemble is a Deep Convolutional Neural Network (DCNN) designed to capture spatial features in the data by a series of convolutional and pooling layers. The CNN processes the input data ${\mathbf{X}}_{c}$ through multiple layers of convolution, denoted as ${C}_{1}$ and ${C}_{2}$, followed by pooling layers ${P}_{1}$ and ${P}_{2}$. The final FS, ${\mathbf{z}}_{\text{CNN}}$, is compressed and passed to a Fully Connected (FC) layer for prediction.

The second base learner, CNN + LSTM, extends CNN’s capabilities by incorporating LSTM layers. The LSTM layers are designed to model temporal dependencies in the data, capturing long-term patterns and trends. The CNN processes the spatial features of the input data as labelled earlier, and the resulting feature maps are input into the LSTM layers. These layers generate a temporal FS, ${\mathbf{z}}_{\text{LSTM}}$, which is compressed and connected to an FC layer for prediction.

The third base learner, CNN + GRU, provides an alternative method to temporal modelling by replacing LSTM with GRU. GRUs are computationally more efficient than LSTMs and retain the ability to capture temporal dependencies. Like the CNN + LSTM, the GRU layers process the CNN spatial FE. The temporal FS, ${\mathbf{z}}_{\text{GRU}}$, is compressed and passed using an FC layer to produce predictions.

Once the predictions from the base learners are generated, they are aggregated by the mea-learner, a QR-RF. The QR-RF combines the strengths of Decision Trees (DT) and Quantile Regression (QR) to provide predictions and uncertainty quantification. The inputs to the QR-RF are the predictions from the base learners, ${\mathbf{z}}_{\text{CNN}},{\mathbf{z}}_{\text{LSTM}},{\mathbf{z}}_{\text{GRU}}$, and the output is the final effluent quality prediction ${\mathbf{\hat{Y}}}$.

##### Base learning models

(i) CNN model for effluent quality prediction
  The $\text{CNN}$ processes the input matrix ${\mathbf{X}}_{c}\in {\mathbb{R}}^{T\times F}$, where ${\prime}T{\prime}$ represents the number of time steps and ${\prime}F{\prime}$ the number of features. The primary objective of the CNN is to extract high-level spatial features that can be used for effluent quality prediction. The CNN begins with an input layer that accepts the matrix ${\mathbf{X}}_{c}$. A sequence of convolutional layers passes this matrix, each followed by an activation function and pooling layer. The first convolutional layer applies ${K}_{1}=32$ filters, each of size $3\times 3$, with a stride of 1.
  The convolution operation is defined as Eq. (1)
  1 $${\mathbf{C}}_{1} = \sigma \left( {{\mathbf{W}}_{1} {*}{\mathbf{X}}_{c} + {\mathbf{b}}_{1} } \right)$$
  where,
  $\mathbf{*}$ → the convolution operation
  ${\mathbf{W}}_{1}$ → the weights of the filters
  $${\mathbf{b}}_{1}$$ → the bias
  $\sigma (\cdot )$→ the activation function, typically $\text{ReLU}$.
  ${\mathbf{C}}_{1}$ → a feature map that highlights spatial patterns in the data.
  Following the first convolutional layer, a max-pooling operation is applied to downsample the feature map and reduce its dimensionality. The max-pooling layer uses a kernel size of $2\times 2$ and a stride of 2, and it is defined as Eq. (2)
  2 $${\mathbf{P}}_{1}=\text{MaxPool}\left({\mathbf{C}}_{1}\right)$$
  where, each $2\times 2$ window in ${\mathbf{C}}_{1}$ is replaced by its maximum value, preserving the most significant features while reducing computational complexity. The second convolutional layer applies ${K}_{2}=64$ filters of size $3\times 3$ to the pooled feature map ${\mathbf{P}}_{1}$, generating a more refined set of features, Eq. (3)
  3 $${\mathbf{C}}_{2} = \sigma \left( {{\mathbf{W}}_{2} {*}{\mathbf{P}}_{1} + {\mathbf{b}}_{2} } \right)$$
  This is followed by a second max-pooling operation, which further reduces the dimensionality, Eq. (4)
  4 $${\mathbf{P}}_{2} = {\text{MaxPool}}\left( {{\mathbf{C}}_{2} } \right)$$
  The output of the second pooling layer, ${\mathbf{P}}_{2}$, is a multi-dimensional tensor containing the extracted spatial features.
  This tensor is compressed into a one-dimensional vector, Eq. (5)
  5 $${\mathbf{z}}_{{{\text{CNN}}}} = {\text{Flatten}}\left( {{\mathbf{P}}_{2} } \right)$$
  which serves as the input to the FC layer.
  The FC layer transforms the feature vector into a prediction for the effluent quality parameter, Eq. (6)
  6 $$\hat{y} = {\mathbf{W}}_{{{\text{fc}}}} \cdot {\mathbf{z}}_{{{\text{CNN}}}} + {\mathbf{b}}_{{{\text{fc}}}}$$
  where ${\mathbf{W}}_{\text{fc}}$ and ${\mathbf{b}}_{\text{fc}}$ are the weights and biases of the FC layer.
  In summary, CNN processes the input time-series data through convolutional and pooling layers to extract spatial features, which are then used to make predictions. The kernel sizes $(3\times 3)$, the number of filters (32 and 64), and the pooling operations are carefully selected to balance FE with computational efficiency.
(ii) CNN + LSTM
  The input to the CNN + LSTM is a time-series matrix, ${\mathbf{X}}_{c}\in {\mathbb{R}}^{T\times F}$, where $T$ represents the number of time steps, and $F$ is the number of features. The CNN processes this input to spatial FE, as described in the standalone CNN section. The compressed output of the CNN, ${\mathbf{z}}_{\text{CNN}}$, is a 1-D vector representing the spatially encoded features. The compressed spatial features ${\mathbf{z}}_{\text{CNN}}$ are reshaped into a time-series format to serve as input to the LSTM layers. The reshaped matrix, ${\mathbf{Z}}_{\text{CNN}}\in {\mathbb{R}}^{{T}^{{^{\prime}}}\times D}$, has ${T}^{{^{\prime}}}$ time steps ($T$) and $D$ features (determined by the CNN output). This reshaping ensures that the temporal dimension is preserved for further processing by the LSTM.
  The LSTM layers process the sequential features ${\mathbf{Z}}_{\text{CNN}}$, capturing temporal dependencies across the time steps. Each LSTM layer maintains an internal and hidden cell state, updated at each time step.
  The LSTM computation at time step $t$ is defined as Eq. (7) to Eq. (11)
  7 $${\mathbf{f}}_{t} = \sigma \left( {{\mathbf{W}}_{f} {\mathbf{x}}_{t} + {\mathbf{U}}_{f} {\mathbf{h}}_{t - 1} + {\mathbf{b}}_{f} } \right)$$
  8 $${\mathbf{i}}_{t} = \sigma \left( {{\mathbf{W}}_{i} {\mathbf{x}}_{t} + {\mathbf{U}}_{i} {\mathbf{h}}_{t - 1} + {\mathbf{b}}_{i} } \right)$$
  9 $${\mathbf{o}}_{t} = \sigma \left( {{\mathbf{W}}_{o} {\mathbf{x}}_{t} + {\mathbf{U}}_{o} {\mathbf{h}}_{t - 1} + {\mathbf{b}}_{o} } \right)$$
  10 $${\mathbf{c}}_{t} = {\mathbf{f}}_{t} \odot {\mathbf{c}}_{t - 1} + {\mathbf{i}}_{t} \odot {\text{tanh}}\left( {{\mathbf{W}}_{c} {\mathbf{x}}_{t} + {\mathbf{U}}_{c} {\mathbf{h}}_{t - 1} + {\mathbf{b}}_{c} } \right)$$
  11 $${\mathbf{h}}_{t} = {\mathbf{o}}_{t} \odot {\text{tanh}}\left( {{\mathbf{c}}_{t} } \right)$$
  where ${\mathbf{f}}_{t},{\mathbf{i}}_{t},{\mathbf{o}}_{t}$ represent the forget, input, and output gates, ${\mathbf{c}}_{t}$ is the cell state, ${\mathbf{h}}_{t}$ is the hidden state, ${\mathbf{x}}_{t}$ is the input at time step $t$, and $\mathbf{W},\mathbf{U},\mathbf{b}$ are trainable weights and biases. The activation functions $\sigma (\cdot )$ and $\text{tanh}(\cdot )$ are the sigmoid and hyperbolic tangent functions. The LSTM layers process the input sequentially, updating the hidden state ${\mathbf{h}}_{t}$ and cell state ${\mathbf{c}}_{t}$ at each time step. The final hidden state ${\mathbf{h}}_{T}$, corresponding to the last time step, encodes the temporal features of the input data. The final hidden state ${\mathbf{h}}_{T}$ is passed through an FC layer to generate the effluent quality prediction.
  This transformation is defined as Eq. (12)
  12 $$\hat{y} = {\mathbf{W}}_{{{\text{fc}}}} \cdot {\mathbf{h}}_{T} + {\mathbf{b}}_{{{\text{fc}}}}$$
  where ${\mathbf{W}}_{\text{fc}}$ and ${\mathbf{b}}_{\text{fc}}$ are the weights and biases of the FC layer and $\hat{y}$ represents the predicted effluent quality parameter.
(iii) CNN + GRU
  The CNN + GRU is a hybrid DL that combines the spatial FE capabilities of CNN with the temporal modelling merits of GRU. This model is designed to process sequential data, capturing spatial patterns from the input features and temporal dependencies across time steps. The CNN + GRU is particularly well-suited for applications in WWTCS, where time-series sensor data is critical for accurate effluent quality prediction. The input to the CNN + GRU is a time-series matrix, ${\mathbf{X}}_{c}\in {\mathbb{R}}^{T\times F}$, where $T$ denotes the number of time steps, and $F$ represents the number of features. The CNN layers process this matrix to spatial FE, as the standalone CNN describes. The output of the CNN, ${\mathbf{z}}_{\text{CNN}}$, is a compressed vector containing the spatially encoded features.
  The compressed spatial features ${\mathbf{z}}_{\text{CNN}}$ are reshaped into a sequence format suitable for the GRU layers. The reshaped matrix, ${\mathbf{Z}}_{\text{CNN}}\in {\mathbb{R}}^{{T}^{{^{\prime}}}\times D}$, where ${T}^{{^{\prime}}}$ equals $T$ and $D$ is the feature dimension determined by the CNN output, retaining the temporal structure of the data. The GRU layers process the reshaped features to capture temporal dependencies across time steps. GRUs are a type of RNN that proposes computational efficiency and simplicity compared to LSTM.
  The GRU computation at each time step $t$ is defined as follows: Eqs. (13)–(16).
  13 $${\mathbf{z}}_{t} = \sigma \left( {{\mathbf{W}}_{z} {\mathbf{x}}_{t} + {\mathbf{U}}_{z} {\mathbf{h}}_{t - 1} + {\mathbf{b}}_{z} } \right)$$
  14 $${\mathbf{r}}_{t} = \sigma \left( {{\mathbf{W}}_{r} {\mathbf{x}}_{t} + {\mathbf{U}}_{r} {\mathbf{h}}_{t - 1} + {\mathbf{b}}_{r} } \right)$$
  15 $${\tilde{\mathbf{h}}}_{t} = {\text{tanh}}\left( {{\mathbf{W}}_{h} {\mathbf{x}}_{t} + {\mathbf{U}}_{h} \left( {{\mathbf{r}}_{t} \odot {\mathbf{h}}_{t - 1} } \right) + {\mathbf{b}}_{h} } \right)$$
  16 $${\mathbf{h}}_{t} = \left( {1 - {\mathbf{z}}_{t} } \right) \odot {\mathbf{h}}_{t - 1} + {\mathbf{z}}_{t} \odot {\tilde{\mathbf{h}}}_{t}$$
  where ${\mathbf{z}}_{t}$ is the update gate, ${\mathbf{r}}_{t}$ is the reset gate, ${\tilde{\mathbf{h}}}_{t}$ is the candidate’s hidden state, ${\mathbf{h}}_{t}$ is the hidden state at time step $t$, and ${\mathbf{x}}_{t}$ is the input at time step $t$. The parameters $\mathbf{W},\mathbf{U},\mathbf{b}$ are trainable weights and biases, and $\sigma (\cdot )$ and $\text{tanh}(\cdot )$ are the sigmoid and hyperbolic tangent activation functions, respectively. The GRU layers maintain a compact representation of the temporal dynamics using the hidden states. The final hidden state, ${\mathbf{h}}_{T}$, represents the temporal features of the input sequence and is passed to the FC layer. The final hidden state ${\mathbf{h}}_{T}$ from the GRU layers is passed using an FC layer to generate the effluent quality prediction.
  The transformation is given by Eq. (17)
  17 $$\hat{y} = {\mathbf{W}}_{{{\text{fc}}}} \cdot {\mathbf{h}}_{T} + {\mathbf{b}}_{{{\text{fc}}}}$$
  where ${\mathbf{W}}_{\text{fc}}$ and ${\mathbf{b}}_{\text{fc}}$ are the weights and biases of the FC layer and $\hat{y}$ represents the predicted effluent quality parameter.

##### Meta learner

(i) Quantile regression
  Quantile Regression (QR) is a statistical technique that estimates conditional quantiles ${Q}_{\tau }(Y\mid \mathbf{X})$ of a target variable ${\prime}Y{\prime}$ given predictors $\mathbf{X}$. This approach generalizes linear regression, approximating the conditional mean ${\mathbb{E}}[Y\mid \mathbf{X}]$, to allow for more detailed knowledge of the distribution of ${\prime}Y{\prime}$ by modelling specific quantiles ${\prime}\tau {\prime}$, where $\tau \in (0,1)$.
  The conditional quantile ${Q}_{\tau }(Y\mid \mathbf{X})$ is defined as, Eq. (18)
  18 $${Q}_{\tau }(Y\mid \mathbf{X})=\text{Inf}\{q\in {\mathbb{R}}:F(q\mid \mathbf{X})\ge \tau \}$$
  where $F(q\mid \mathbf{X})$ is the conditional cumulative Distribution Function (CDF) of $Y$ assumed $\mathbf{X}$. Intuitively, ${Q}_{\tau }(Y\mid \mathbf{X})$ is the value of $Y$ below which $100\tau \text{\%}$ of observations fall.
  To compute ${Q}_{\tau }(Y\mid \mathbf{X})$, quantile regression minimizes the following objective function, Eq. (19).
  19 $$\mathop {{\text{Min}}}\limits_{\beta } \mathop \sum \limits_{i = 1}^{n} \rho_{\tau } \left( {y_{i} - {\mathbf{X}}_{i}^{{ \top }} \beta } \right)$$
  where ${\mathbf{X}}_{i}^{{\top }}\beta$ is the predicted value for the $i$-th observation, ${y}_{i}$ is the actual value and ${\rho }_{\tau }(u)$ is the quantile loss function, Eq. (2).
  20 $$\rho_{\tau } \left( u \right) = \left\{ {\begin{array}{*{20}l} {\tau u,} \hfill & {u \ge 0} \hfill \\ {\left( {1 - \tau } \right)\left( { - u} \right),} \hfill & {u < 0} \hfill \\ \end{array} } \right.$$
  The quantile loss function ${\rho }_{\tau }(u)$ penalizes overestimation and underestimation differently, with weights ${\prime}\tau {\prime}$ and $1-\tau$. Solving this optimization problem generates the regression coefficients ${\prime}\beta {\prime}$, which define the linear relationship between $\mathbf{X}$ and the quantile ${Q}_{\tau }(Y\mid \mathbf{X})$.
(ii) QR-RF
  QR-RF extends random forests to predict conditional quantiles of ${\prime}Y{\prime}$. The RF is an ensemble of $M$, and DT as ${T}_{1},{T}_{2},\dots ,{T}_{M}$, each trained on a bootstrapped subset of the training data. Traditional RF aggregate tree predictions by averaging; in QR-RF, the trees are designed to predict conditional distributions, enabling quantile prediction.
  Each DT in the forest partitions the input space into regions ${R}_{1},{R}_{2},\dots ,{R}_{L}$, where $L$ is the number of leaf nodes in the tree. For an assumed leaf ${R}_{l}$, the set of target values for samples falling into this leaf is $\left\{{y}_{i}:{\mathbf{X}}_{i}\in {R}_{l}\right\}$. Let ${\mathbf{z}}_{\text{Combined}}$ be the input feature vector for QR-RF.
  For a given tree ${T}_{m}$, the target distribution at a leaf ${R}_{l}$ is represented as, Eq. (21)
  21 $$F\left( {q{\mid }{\mathbf{z}}_{{\text{Combined }}} } \right) = \frac{1}{{\left| {R_{l} } \right|}}\mathop \sum \limits_{{y_{i} \in R_{l} }} {\mathbb{I}}\left( {y_{i} \le q} \right)$$
  where ${\mathbb{I}}(\cdot )$ is the indicator function that equals 1 if ${y}_{i}\le q$ and 0 otherwise. This empirical CDF is used to estimate quantiles for the leaf. To predict the $\tau$-th quantile for a new input ${\mathbf{z}}_{\text{Combined,}}$ QR RF aggregates the empirical distributions from all $M$ trees, Eq. (22)
  The $\tau$-th quantile is computed as Eq. (22)
  22 $$\hat{Q}_{\tau } \left( {Y{\mid }{\mathbf{z}}_{{\text{combined }}} } \right) = \frac{1}{M}\mathop \sum \limits_{m = 1}^{M} \hat{Q}_{\tau }^{\left( m \right)} \left( {Y{\mid }{\mathbf{z}}_{{\text{combined }}} } \right)$$
  where ${\hat{Q}}_{\tau }^{(m)}\left(Y\mid {\mathbf{z}}_{\text{combined}}\right)$ is the quantile approximation from the $m$-th tree.
  This aggregation ensures robustness and reduces variance in the predictions. During DT construction, QR RF selects splits based on variance reduction. For a parent node ${R}_{p}$ split into left and right child nodes ${R}_{\text{left}}$ and ${R}_{\text{right,}}$, the variance reduction is assumed by Eq. (23)
  23 $${{\Delta Var}} = {\text{Var}}\left( {Y_{{R_{p} }} } \right) - \frac{{\left| {R_{{\text{left }}} } \right|}}{{\left| {R_{p} } \right|}}{\text{Var}}\left( {Y_{{R_{{\text{left }}} }} } \right) - \frac{{\left| {R_{{\text{right }}} } \right|}}{{\left| {R_{p} } \right|}}{\text{Var}}\left( {Y_{{R_{{\text{right }}} }} } \right),$$
  where $|R|$ denotes the number of samples in region $R$, and $\text{Var}\left({Y}_{R}\right)$ is the variance of $Y$ within region $R$. This criterion ensures that each split minimizes the uncertainty in the target variable.
(iii) QR-RF as meta-learner for ensemble predictions
  QR-RF acts as a meta-learner in this work, aggregating predictions from the base learners-Deep CNN, CNN + LSTM, and CNN + GRU. Let ${\mathbf{z}}_{\text{CNN}},{\mathbf{z}}_{\text{LSTM}}$, and ${\mathbf{z}}_{\text{GRU}}$ represent the FS from these models.

These are concatenated to form the input to the QR-RF, Eq. (24)

24 $${\mathbf{z}}_{\text{combined }}=\left[{\mathbf{z}}_{\text{CNN}},{\mathbf{z}}_{\text{LSTM}},{\mathbf{z}}_{\text{GRU}}\right]$$

Assumed the combined features ${\mathbf{z}}_{\text{combined,}}$ QR-RF predicts point estimates and quantile-based uncertainty bounds for the effluent quality parameter $Y$.

The outputs include:

1. Point Prediction (Median): ${\hat{y}}_{\text{median }}={\hat{Q}}_{0.50}\left(Y\mid {\mathbf{z}}_{\text{combined}}\right)$
2. Uncertainty Bounds (*e.g.,* 90% confidence interval): $\left[{\hat{Q}}_{0.05}\left(Y\mid {\mathbf{z}}_{\text{combined }}\right),{\hat{Q}}_{0.95}\left(Y\mid {\mathbf{z}}_{\text{combined}}\right)\right]$

The QR-RF meta-learner leverages its ability to model conditional distributions, ensuring robust predictions while quantifying uncertainty. This combination of robustness and interpretability is critical for effluent quality prediction in dynamic and uncertain environments such as WWTCS.

In wastewater treatment systems, the characteristics of incoming influents, including pollutant concentrations such as COD, TSS, NH₄⁺-N, and variations in flow rates and pH, exhibit high temporal variability and non-linear dynamics. These fluctuations, driven by changing industrial loads, residential discharges, and environmental factors (*e.g.,* rainfall), create significant challenges for predictive modelling. Accurate prediction of effluent quality becomes difficult when the system must respond to abrupt shifts in influent features, leading to increased prediction uncertainty and potential operational risks.

The proposed ensemble DL, incorporating CNN, CNN + LSTM, and CNN + GRU base learners with a QR-RF meta-learner, explicitly addresses these complexities by capturing spatial–temporal dependencies and providing uncertainty-aware predictions. The predicted outputs, including estimated COD, TSS, and NH₄⁺-N concentrations with confidence intervals, feed directly into the ICS, enabling dynamic adjustments in chemical dosing rates, aeration intensities, and sludge recirculation strategies. This predictive control loop ensures that treatment objectives are maintained despite the inherent variability in influent quality, optimizing operational efficiency while ensuring regulatory compliance for effluent discharge.

## Case study: application to Jiangsu municipal WWTP

### Study area description

The case study was conducted at the Jiangsu Municipal WWTCS in Suzhou Industrial Park, Jiangsu Province, China. This facility represents a large-scale municipal WWTP with a designed daily treatment capacity of 200,000 cubic meters, serving a mix of industrial and residential influent sources. The treatment process encompasses stages, including preliminary mechanical treatment, biological oxidation, chemical coagulation, sedimentation, and sludge management operations. The facility is operated under strict compliance with Chinese environmental discharge standards (GB 18,918–2002), ensuring that effluent quality parameters, such as Chemical Oxygen Demand (COD), Biochemical Oxygen Demand (BOD), Total Suspended Solids (TSS), and ammonia nitrogen, meet regulatory thresholds before discharge. To support modern monitoring and control requirements, the plant has implemented an extensive instrumentation system based on IoT-enabled sensor networks strategically deployed across key points of the wastewater treatment process.

### Data collection and processing

Experimental data for this study were collected over a continuous 24-month from January 2022 to December 2023. The plant’s monitoring infrastructure comprises 86 IoT sensors distributed across numerous operational units, including influent channels, aeration tanks, secondary clarifiers, sludge thickeners, and effluent discharge points. These sensors continuously recorded key operational parameters such as flow rates, temperature, pressure, liquid levels, pH, and dissolved oxygen concentrations. The systematic deployment and high-frequency acquisition from these devices generated a comprehensive dataset exceeding one million data points, capturing both routine operational dynamics and occasional disturbances.

To ensure the protection of sensitive operational and infrastructural information, a rigorous data anonymization protocol was employed. All collected data were pre-processed to remove identifiers and encoded following the GB/T 35,273–2020 standard for personal data security. Additionally, the entire data collection and storage procedure adhered to national environmental monitoring standards (GB 18,918–2002) and aligned with international best practices for data protection. The resulting dataset provided a robust and accurate foundation for training, validating, and testing the proposed ensemble DL and control system framework.

### Dataset description

The compiled dataset from the Jiangsu Municipal WWTCS encompassed a broad range of operational and environmental variables recorded over a continuous 24-month monitoring period. Data were captured from 86 IoT sensors installed across critical stages of the wastewater treatment process. Each sensor type monitored specific parameters relevant to process control, effluent quality assessment, and operational optimization. The dataset included high-frequency time-series measurements with varying sampling intervals depending on the criticality of the parameter monitored.

The dataset contained approximately 57.88 million data points, providing a vibrant temporal feature of model behavior under routine operating conditions and transient events. The variables collected, their types, units of measurement, and typical sampling intervals are summarized in Table 2. All data underwent severe preprocessing steps, including outlier removal, missing value imputation via forward-filling, normalization, and encoding sensitive operational variables to ensure data quality and regulatory compliance before model training and evaluation.

**Table 2:** Overview of dataset variables.

No	Parameter	Sensor type	Unit	Sampling interval	Description
1	Flow rate	Ultrasonic flowmeter	m^3^/h	1 min	Influent and effluent flow monitoring
2	Water temperature	Thermistor	°C	5 min	Temperature monitoring at different process stages
3	Pressure	Pressure transducer	kPa	1 min	Monitoring aeration tank and sludge line pressures
4	Liquid level	Radar level sensor	meters	1 min	Liquid level monitoring in tanks and clarifiers
5	pH	pH electrode	pH units	5 min	Monitoring acidity/alkalinity at multiple stages
6	Dissolved oxygen (DO)	Optical DO sensor	mg/L	1 min	Aeration tank and biological treatment monitoring
7	Chemical oxygen demand (COD)	Online analyzer	mg/L	15 min	COD concentrations in reactors and effluent
8	Total suspended solids (TSS)	Optical TSS probe	mg/L	15 min	TSS levels in clarifiers and effluent streams
9	Ammonia nitrogen (NH₄⁺-N)	Ion selective electrode	mg/L	15 min	Ammonia nitrogen concentrations in treated effluent
10	Sludge volume index (SVI)	Manual sampling	mL/g	1 day	Characterizing sludge-settling properties

The two-year dataset (January 2022–December 2023) was specifically selected to ensure comprehensive coverage of seasonal patterns, operational dynamics, and transient conditions inherent to large-scale wastewater treatment operations. Shorter observation periods would inadequately represent the full variability required for building a robust, generalizable predictive model. The input parameters utilized in the predictive analytics — flow rate, water temperature, pressure, liquid level, pH, dissolved oxygen (DO), COD, TSS, ammonia nitrogen (NH₄⁺-N), and sludge volume index (SVI) — were selected based on their direct operational relevance to treatment efficiency, effluent quality, and regulatory compliance. These parameters are scientifically critical for biological and chemical process monitoring and practically available using real-time sensor measurements, making them suitable for integration into the IoT-enabled predictive control model.

The influent processed at Jiangsu Municipal WWTCS constitutes a mixed wastewater stream comprising domestic sewage and industrial effluents. Industrial contributors include light manufacturing, electronics, food processing, and chemical production facilities. This mixed composition provides a realistic and operationally complex dataset for testing the predictive and control capabilities of the proposed framework.

### Model training and implementation

All predictive modelling and control system components were implemented in Python 3.8, utilizing TensorFlow 2.7, Keras API, Scikit-learn 1.0.2 for DL and ensemble modelling, and Pandas and NumPy libraries for data preprocessing. The QR-RF meta-learner was developed using a customized ensemble wrapper based on SCI-KIT-Learn’s RandomForestRegressor class with quantile loss modifications. Training and inference computations were executed on a high-performance computing server with NVIDIA Tesla V100 GPU (32 GB memory), Intel Xeon Gold 6248 CPU @ 2.50 GHz, and 256 GB RAM. GPU acceleration was employed for DL training, while CPU resources were used for random forest computations and data handling tasks.

The compiled dataset was partitioned into three subsets for model training and evaluation: 70% for training, 15% for validation, and 15% for testing. The partitioning was performed using a chronological split rather than random sampling to respect the inherent temporal dependencies of the wastewater treatment time-series data. Specifically, the first 70% of the time-ordered records were used for model training, the next 15% for validation during hyperparameter tuning, and the final 15% for independent testing of model generalization. This time-based partitioning approach prevents information leakage across temporal boundaries and simulates realistic operational deployment scenarios, where future data must be predicted based solely on past observations. Early stopping based on validation loss was employed to mitigate overfitting during training.

This study implemented three convolutional layers for the CNN with 32, 64, and 128 filters, each using a kernel size 3 × 3 and ReLU activation. Max pooling layers with pool size 2 × 2 were presented between convolutional layers. The final dense layer contained 256 neurons with ReLU activation, followed by a dropout rate of 0.3 to prevent overfitting.

The learning rate was set to 0.001 using the Adam optimizer, and the model was trained for 100 epochs with a batch size of 64. The hybrid CNN + LSTM extended the CNN by adding two LSTM layers with 128 and 64 units—the LSTM layers employed *tanh* activation functions with recurrent dropout set to 0.2. The sequence length was configured to 24-time steps, corresponding to a full day of measurements at hourly intervals. Due to its increased complexity, the model maintained the same learning rate and optimizer as the CNN but required 150 epochs for convergence. The CNN + GRU followed a similar model, replacing the LSTM with GRU layers of equal sizes (128 and 64 units). The GRU layers applied the same tanh activation function but with a slightly lower recurrent dropout of 0.15, as GRU typically requires less regularization. This model was trained for 130 epochs with the same batch size and learning rate as the previous models. Tables 3 and 4 provide the proposed model and training parameters.

**Table 3:** Model parameters.

Parameter	CNN	CNN + LSTM	CNN + GRU	QR-RF
Input shape	(24, 12)	(24, 12)	(24, 12)	N/A
Number of features	12	12	12	Combined predictions
Convolutional layers	3	3	3	N/A
Conv filters	32, 64, 128	32, 64, 128	32, 64, 128	N/A
Kernel size	3 × 3	3 × 3	3 × 3	N/A
Pooling size	2 × 2	2 × 2	2 × 2	N/A
LSTM/GRU units	N/A	128, 64	128, 64	N/A
Dense layer units	256	256	256	N/A
Number of trees	N/A	N/A	N/A	500

**Table 4:** Training parameters.

Parameter	CNN	CNN + LSTM	CNN + GRU	QR-RF
Batch Size	64	64	64	N/A
Learning rate	0.001	0.001	0.001	N/A
Epochs	150	150	150	N/A
Dropout rate	0.3	0.3	0.3	N/A
Recurrent dropout	N/A	0.2	0.15	N/A
Early stopping patience	15	15	15	N/A
LR reduction factor	0.1	0.1	0.1	N/A
LR reduction patience	10	10	10	N/A
Min samples per leaf	N/A	N/A	N/A	10
Max tree depth	N/A	N/A	N/A	20

For all neural network models, this work implemented early stopping with patience of 15 epochs, monitoring the validation loss and learning rate reduction on the plateau with a factor of 0.1 and patience of 10 epochs. The loss function employed was Mean Squared Error (MSE) for the base models, while the QR-RF meta-learner optimized quantile loss at multiple quantile levels (0.1, 0.25, 0.5, 0.75, 0.9). The QR-RF meta-learner was configured with 500 trees, each with a maximum depth of 20 and a minimum of 10 samples per leaf node. Following standard RF practices, the number of features considered for each split was set to the square root of the sum of features. The model applied bootstrap sampling with out-of-bag error estimation for continuous performance monitoring. The training was conducted on a high-performance computing cluster equipped with NVIDIA Tesla V100 GPUs, utilizing distributed training capabilities to control big data efficiently. The total training time was approximately 48 h for all models combined, with the CNN + LSTM requiring the most prolonged training period due to its model complexity. The trained models were exported using the TensorFlow SavedModel format for the neural network components and fixed serialization for the QR-RF meta-learner. The complete model pipeline, including preprocessing steps and prediction logic, was containerized using Docker to ensure consistent deployment across different environments. The preprocessing steps included standardizing numerical features, handling missing values by forward-fill methods, and sequence padding for temporal models.

### Design of ICS

**Fig. 13:**
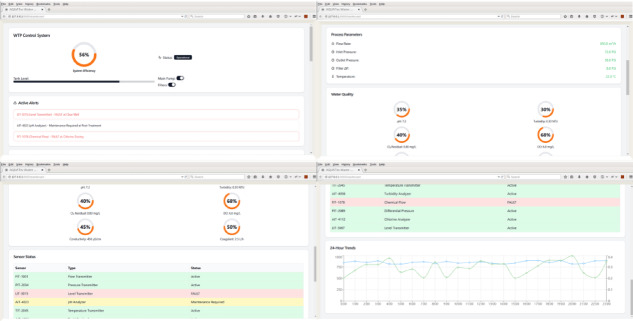
AQUAtec ICS screenshot.

The AQUAtec ICS (Fig. 13) is implemented using *React.js* for the frontend interface and *Node.js* for backend operations, with WebSocket protocol enabling real-time data communication. The system utilizes the Material-UI model for interface components and *Chart.js* for data visualization. The ICS has three main components: process monitoring, quality control, and system maintenance management. The process monitoring interface is developed using React components that display real-time operational parameters. The main control panel implements circular measure components for visualizing system efficiency metrics, with WebSocket connections maintaining continuous updates of flow rates, pressure readings, and treatment parameters. The control panel incorporates Toggle components for equipment operation, connected to *Node.js* backend services that interface with Programmable Logic Controllers (PLCs) through Modbus TCP/IP protocol.

The quality control module processes data from multiple sensor inputs, displaying parameters through a standardized interface. Each parameter (pH, turbidity, chlorine residual, dissolved oxygen) is monitored through dedicated control loops implemented in the backend. The system employs PID controllers programmed in *Node.js*, adjusting tuning parameters based on historical performance data. Industrial sensors implement control actions, with feedback mechanisms ensuring precise chemical dosing and aeration system regulation. System maintenance management is implemented through a database-driven alert system using MongoDB for event logs and PostgreSQL for time-series data storage. The alert mechanism processes sensor status data by predefined threshold algorithms, generating notifications based on operational parameters and equipment status. The sensor status panel uses a RESTful API model to communicate with the plant’s Distributed Control System (DCS), providing real-time status updates for all devices.

The trend analysis functionality is made using the *Chart.js* library, implementing time-series visualization of operational data. The backend maintains a rolling 24-h data buffer in the Redis cache, with longer-term storage managed through TimescaleDB. Data sampling rates are configured according to parameter criticality, with critical parameters sampled at 1-s intervals and less critical at 5-min intervals. ICS security is implemented by JWT (JSON Web Token) authentication and role-based access control, with all communications encrypted using TLS 1.3 protocol. The system operates on a segregated industrial network, with firewalled connections to the plant’s business network for reporting purposes. A redundant storage system maintains regular automated backups of configuration data and control parameters. The control logic incorporates fail-safe mechanisms programmed in the PLC layer, ensuring safe operation even in communication failure with the supervisory system. Emergency shutdown procedures are implemented through hardwired safety circuits, operating independently of the software control system.

## Results and analysis

### Prediction accuracy metrics

**Fig. 14:**
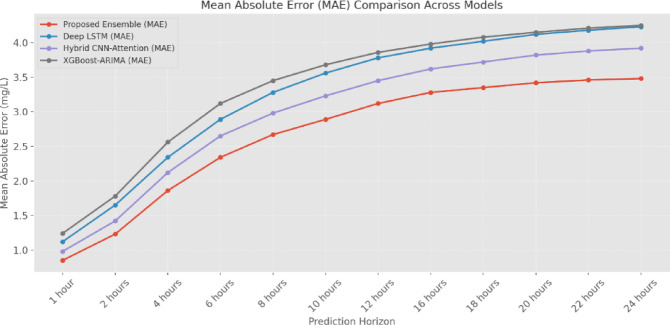
MAE comparison across models (mg/L).

**Fig. 15:**
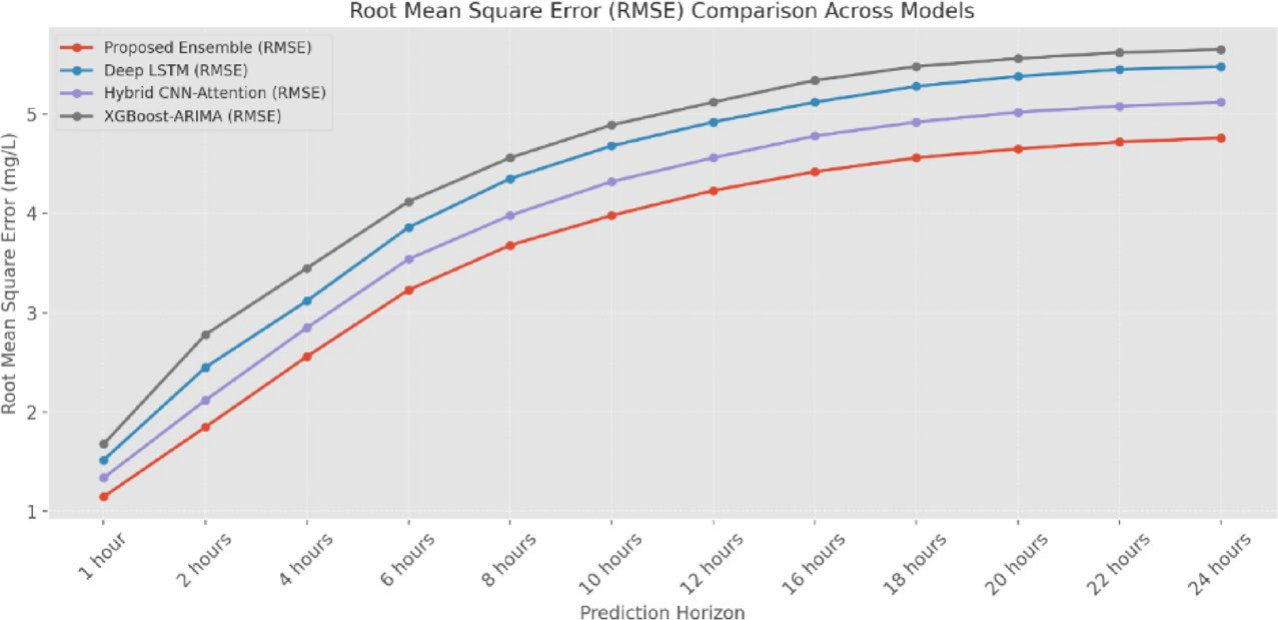
RMSE comparison (mg/L).

The MAE (Fig. 14 and Table 5) analysis demonstrates that the proposed Ensemble Model (EM) consistently outperforms other models across all prediction horizons. At the 1-h prediction horizon, the EM achieves an MAE of 0.85 mg/L, showing a 24% improvement over the Deep-LSTM (1.12 mg/L) and a 31% improvement over the XGBoost-ARIMA approach (1.24 mg/L). This performance advantage is maintained as the prediction horizon extends, with the EM showing an MAE of 3.48 mg/L at 24 h compared to 4.23 mg/L for Deep-LSTM and 4.25 mg/L for XGBoost-ARIMA. The RMSE analysis (Fig. 15 and Table 6) further validates the superior performance of the ensemble approach. For short-term predictions (1-h horizon), the EM achieves an RMSE of 1.15 mg/L, significantly lower than the Hybrid CNN-Attention (1.34 mg/L) and XGBoost-ARIMA (1.68 mg/L) models. The performance gap widens at longer prediction horizons, with the EM maintaining an RMSE of 4.76 mg/L at 24 h, compared to 5.48 mg/L for Deep-LSTM and 5.65 mg/L for XGBoost-ARIMA.

**Table 5:** MAE comparison across models (mg/L).

Prediction horizon	Proposed ensemble	Deep LSTM	Hybrid CNN-attention	XGBoost-ARIMA
1 Hour	0.85	1.12	0.98	1.24
2 Hours	1.23	1.65	1.42	1.78
4 Hours	1.86	2.34	2.12	2.56
6 Hours	2.34	2.89	2.65	3.12
8 Hours	2.67	3.28	2.98	3.45
10 Hours	2.89	3.56	3.23	3.68
12 Hours	3.12	3.78	3.45	3.86
16 Hours	3.28	3.92	3.62	3.98
18 Hours	3.35	4.02	3.72	4.08
20 Hours	3.42	4.12	3.82	4.15
22 Hours	3.46	4.18	3.88	4.21
24 Hours	3.48	4.23	3.92	4.25

**Table 6:** RMSE Comparison (mg/L).

Prediction horizon	Proposed ensemble	Deep-LSTM	Hybrid CNN-attention	XGBoost-ARIMA
1 Hour	1.15	1.52	1.34	1.68
2 Hours	1.85	2.45	2.12	2.78
4 Hours	2.56	3.12	2.85	3.45
6 Hours	3.23	3.86	3.54	4.12
8 Hours	3.68	4.35	3.98	4.56
10 Hours	3.98	4.68	4.32	4.89
12 Hours	4.23	4.92	4.56	5.12
16 Hours	4.42	5.12	4.78	5.34
18 Hours	4.56	5.28	4.92	5.48
20 Hours	4.65	5.38	5.02	5.56
22 Hours	4.72	5.45	5.08	5.62
24 Hours	4.76	5.48	5.12	5.65

**Fig. 16:**
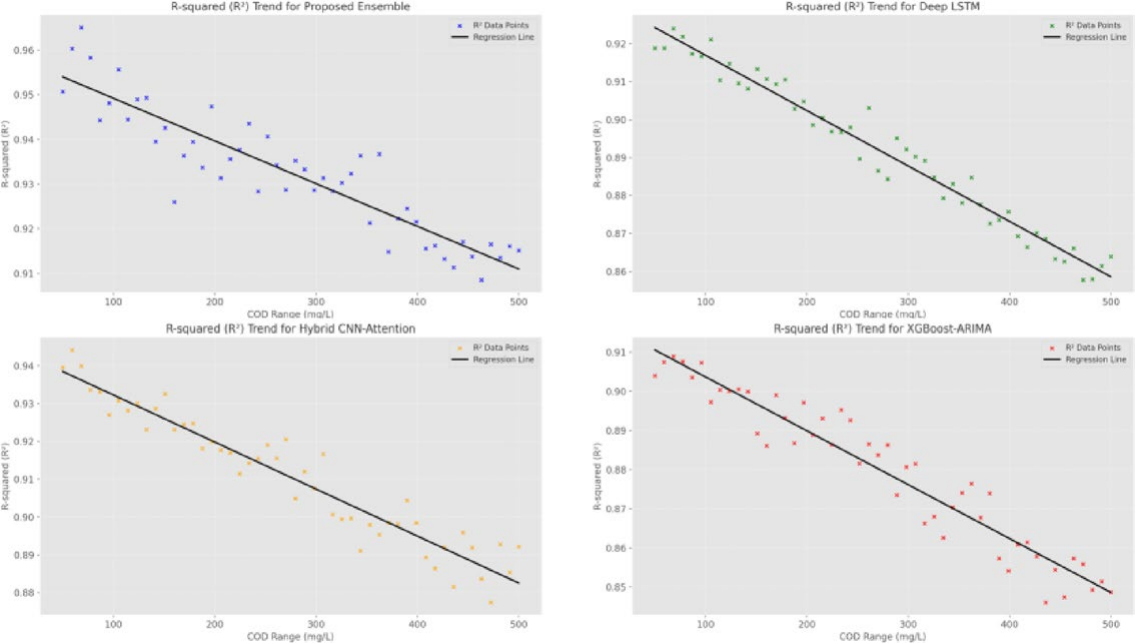
R^2^: Predicted vs. actual cod values.

**Fig. 17:**
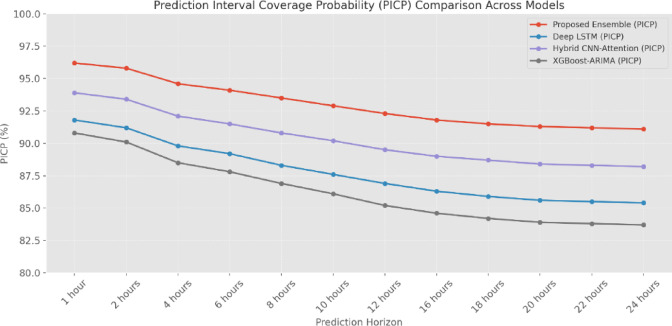
Prediction interval coverage probability (PICP).

The R-squared (Fig. 16 and Table 7) analysis across different COD concentration ranges reveals the EM’s robust performance across variable waste-water conditions. In the lower concentration range (0–100 mg/L), the EM achieves an R^2^ of 0.957, compared to 0.923 for Deep-LSTM and 0.912 for XGBoost-ARIMA. This superior performance is maintained even at higher concentrations (> 400 mg/L), where the EM achieves an R^2^ of 0.912, significantly higher than other models. The Prediction Interval Coverage Probability (Fig. 17 and Table 8) analysis demonstrates the EM’s superior uncertainty quantification capabilities. At the 1-h horizon, the EM achieves a PICP of 96.2%, compared to 91.8% for Deep-LSTM and 90.8% for XGBoost-ARIMA. This advantage in uncertainty assessment is maintained across longer prediction horizons, with the EM showing a PICP of 91.1% at 24 h, compared to 85.4% for Deep-LSTM and 83.7% for XGBoost-ARIMA, indicating more reliable uncertainty limits for operational decision-making.

**Table 7:** R-squared (R^2^) values: predicted vs. actual COD values.

Model	0–100 mg/L	101–200 mg/L	201–300 mg/L	301–400 mg/L	> 400 mg/L
Proposed EM	0.957	0.943	0.935	0.928	0.912
Deep-LSTM	0.923	0.908	0.894	0.882	0.865
Hybrid CNN-Attention	0.938	0.925	0.912	0.901	0.886
XGBoost-ARIMA	0.912	0.895	0.883	0.871	0.854

**Table 8:** Prediction interval coverage probability (PICP).

Prediction horizon	Proposed EM (%)	Deep-LSTM (%)	Hybrid CNN-attention (%)	XGBoost-ARIMA (%)
1 Hour	96.2	91.8	93.9	90.8
2 Hours	95.8	91.2	93.4	90.1
4 Hours	94.6	89.8	92.1	88.5
6 Hours	94.1	89.2	91.5	87.8
8 Hours	93.5	88.3	90.8	86.9
10 Hours	92.9	87.6	90.2	86.1
12 Hours	92.3	86.9	89.5	85.2
16 Hours	91.8	86.3	89.0	84.6
18 Hours	91.5	85.9	88.7	84.2
20 Hours	91.3	85.6	88.4	83.9
22 Hours	91.2	85.5	88.3	83.8
24 Hours	91.1	85.4	88.2	83.7

### Performance metrics

**Fig. 18:**
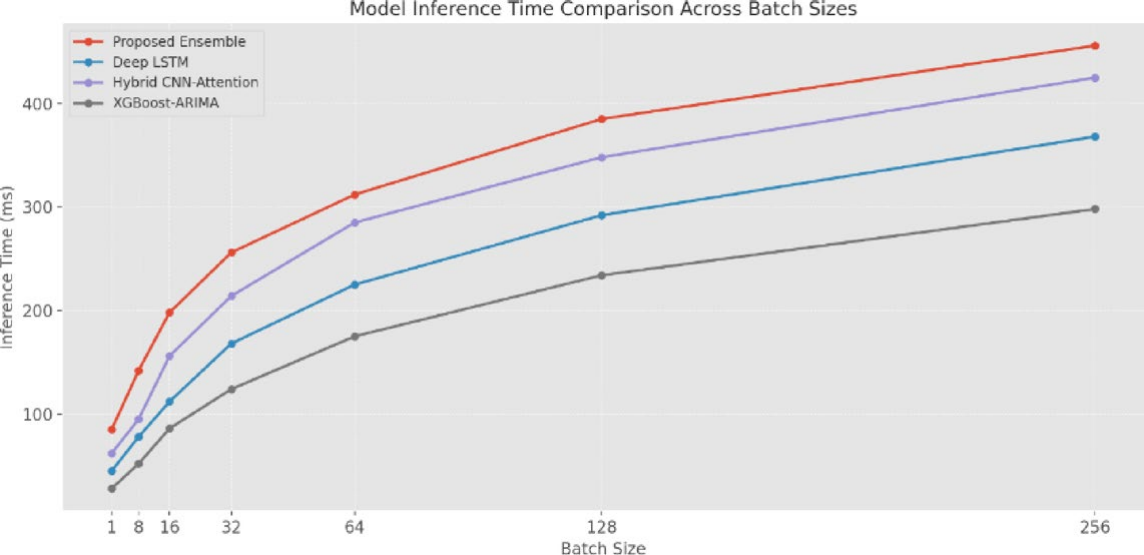
Model inference time comparison (ms).

Examining the inference time (Fig. 18) across different batch sizes has demonstrated that while the proposed EM exhibits higher computational overhead, its performance remains within acceptable bounds for real-time applications. At a batch size of 1, the EM requires 85 ms for inference, compared to 45 ms for Deep-LSTM and 28 ms for XGBoost-ARIMA. This increased latency is attributable to the parallel processing of multiple model components and the meta-learner integration. As batch size increases, all models show expected increases in processing time, with the EM maintaining a relatively consistent overhead ratio. At a batch size of 256, the EM requires 456 ms, while Deep-LSTM and XGBoost-ARIMA require 368 and 298 ms.

**Fig. 19:**
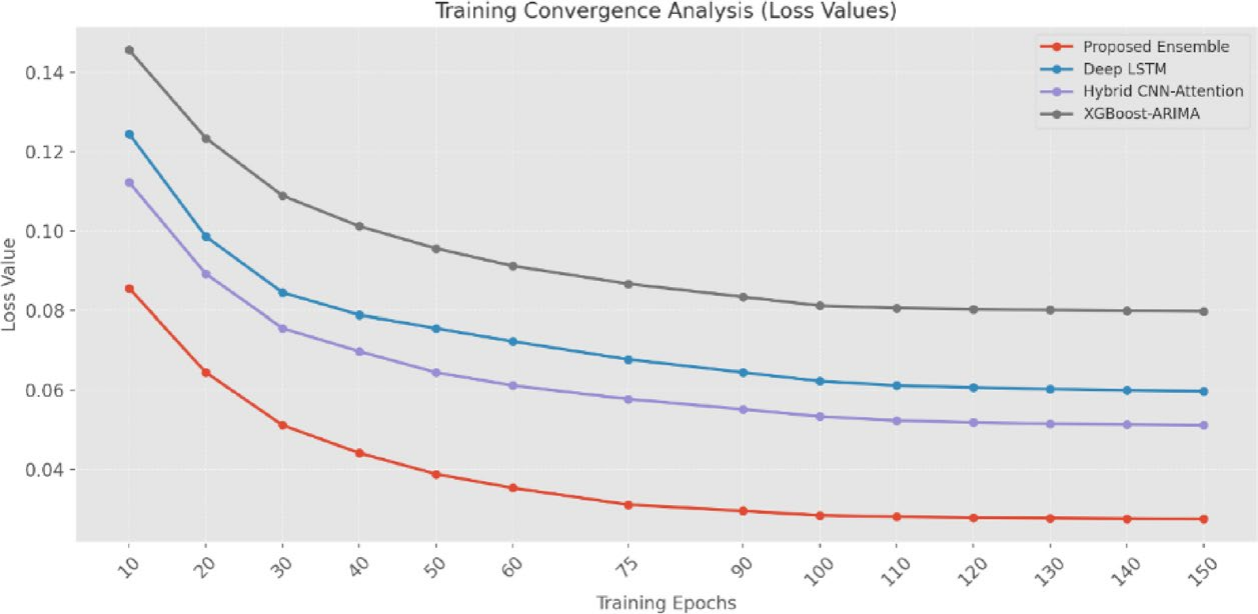
Training convergence analysis (TCA) (loss values).

The TCA (Fig. 19) reveals superior learning dynamics of the ensemble approach. Starting from epoch 10 with a loss value of 0.0856, the EM demonstrates faster convergence than other models. By epoch 50, the EM achieves a loss of 0.0389, significantly lower than Deep-LSTM (0.0756) and XGBoost-ARIMA (0.0956). The convergence pattern continues to show improvement until stabilizing around epoch 120, where the EM reaches a loss of 0.0279. The final converged loss values at epoch 150 (ensemble: 0.0276, Deep-LSTM: 0.0598, Hybrid CNN-Attention: 0.0512, XGBoost-ARIMA: 0.0798) demonstrate that despite higher computational requirements, the EM achieves significantly better optimization.

### Operational metrics

**Fig. 20:**
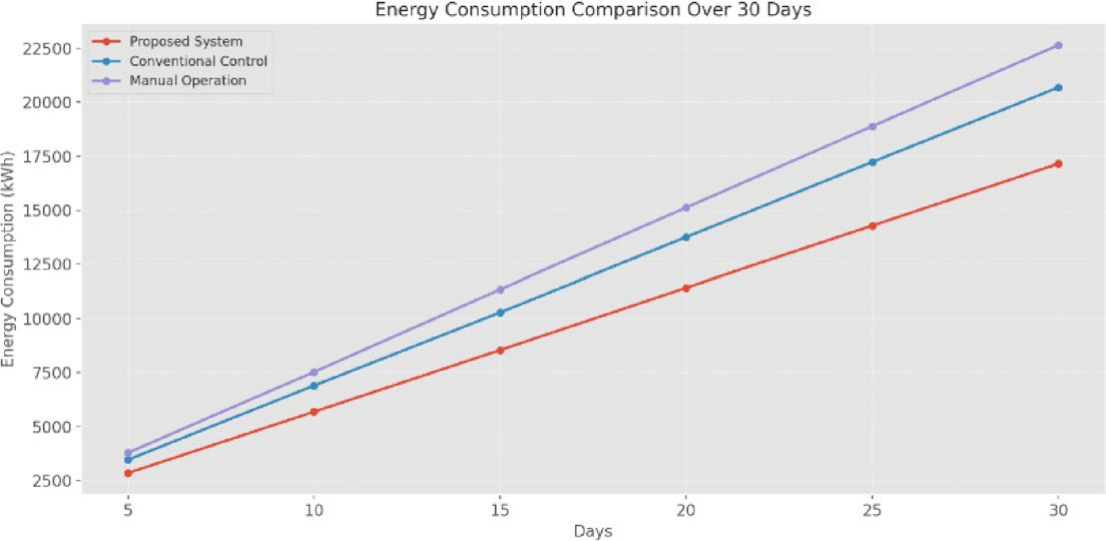
EC comparison (kWh) over 30 days of operation.

Energy Consumption (EC) data (Fig. 20) reveals a systematic reduction in EC, with the proposed system consuming 17,153 kWh after 30 days of operation, compared to 20,687 kWh for conventional control systems and 22,648 kWh for manual operations. This represents a 17% reduction from conventional control methods and a 24% improvement over manual operation.

**Fig. 21:**
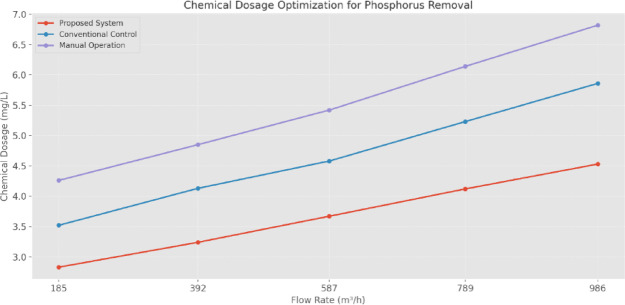
CDO for phosphorus removal.

The CDO (Fig. 21) for phosphorus removal shows precise control capabilities across variable flow rates. At a 185 m^3^/h flow rate, the proposed system maintains effective treatment with a dosage of 2.83 mg/L, compared to 3.52 mg/L for conventional control and 4.26 mg/L for manual operation. This optimization pattern continues across higher flow rates, with the system maintaining proportionally lower chemical usage while achieving treatment targets. At peak flow (986 m^3^/h), the system operates with a CDO of 4.53 mg/L compared to 5.86 mg/L and 6.82 mg/L for conventional and manual operations, demonstrating consistent optimization across operational ranges.

**Fig. 22:**
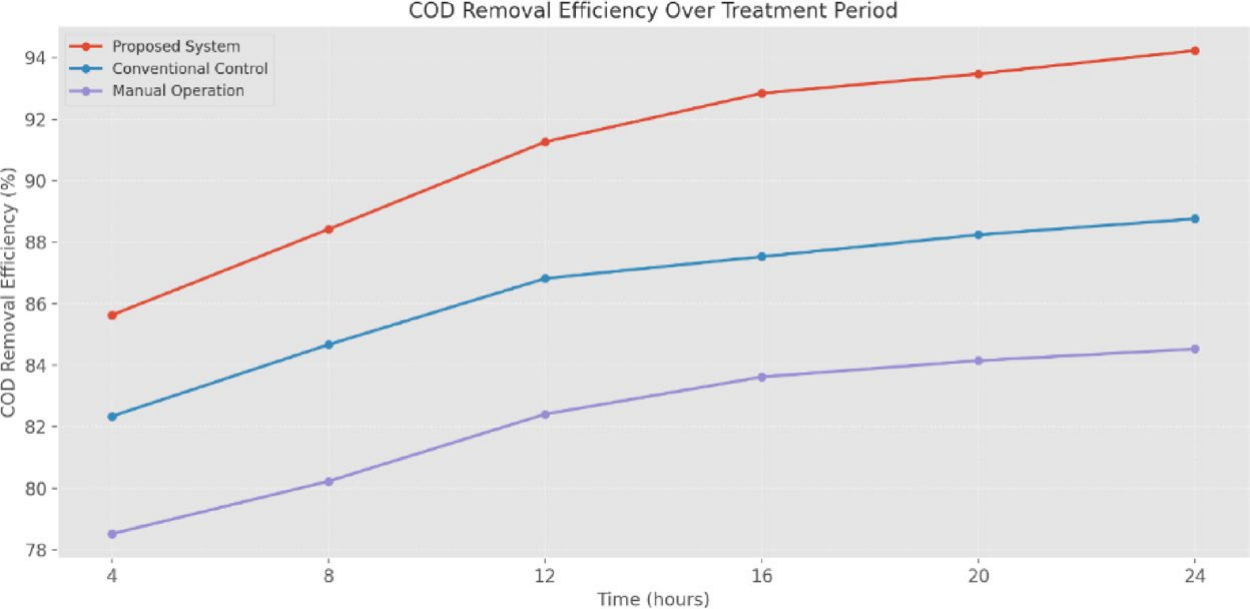
COD removal efficiency over treatment period.

COD removal efficiency analysis (Fig. 22) over a 24-h treatment cycle shows enhanced treatment performance. The proposed system achieves 85.63% removal efficiency within the first 4 h, compared to 82.34% and 78.52% for conventional and manual operations. This performance advantage increases over the treatment period, reaching 94.23% efficiency at 24 h, compared to 88.76% for conventional control and 84.53% for manual operation. The reliable improvement in removal efficiency indicates better process control and optimization of treatment parameters throughout the operational cycle. These results demonstrate that the automated ICS significantly improves operational efficiency by precisely controlling EC, chemical dosage, and treatment processes while maintaining higher treatment standards than conventional approaches.

### Resource utilization metrics

**Fig. 23:**
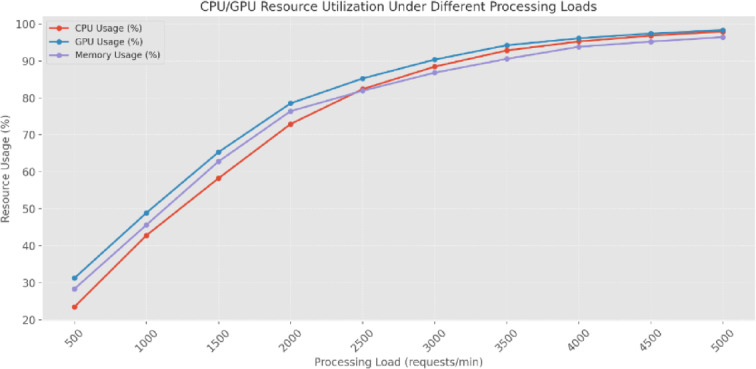
CPU/GPU RU under different processing loads.

The computational Resource Utilization (RU) analysis (Fig. 23) demonstrates the system’s scaling capabilities under variable processing loads. At baseline operations (500 *requests/min*), the system maintains efficient RU with CPU usage at 23.47%, GPU at 31.26%, and Memory Consumption (MC) at 28.34%. As processing demands increase, the system shows linear scaling features up to moderate loads. At 2000 requests/min, RU increases to 72.94% CPU, 78.53% GPU, and 76.42% MC, signifying balanced resource distribution across computing components. The system methods do not exceed resource capacity limits under peak loads (4000–5000 Requests/Min). At 5000 requests/min, CPU utilization reaches 97.94%, GPU usage 98.36%, and MC 96.45%, demonstrating effective resource management even under stress conditions. The parallel utilization patterns of CPU and GPU resources indicate efficient workload distribution between computational components.

**Fig. 24:**
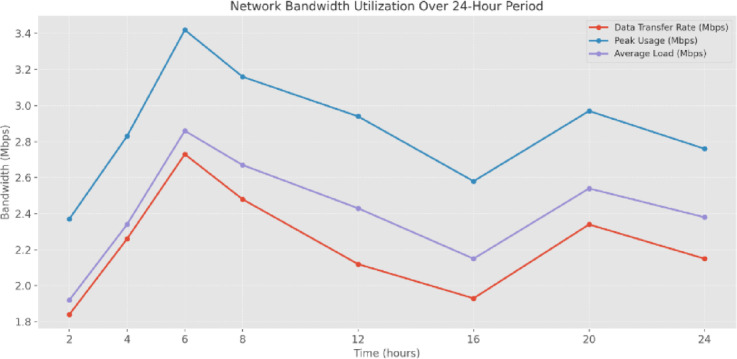
Network bandwidth utilization (NBU) over 24 h.

The analysis of NBU (Fig. 24) over a 24-h operational period reveals cyclical patterns in data transfer requirements. The system maintains an average data transfer rate between 1.84 Mbps and 2.73 Mbps, with peak usage ranging from 2.37 Mbps to 3.42 Mbps. The highest NBU occurs during the 6-h mark, with a peak of 3.42 Mbps, corresponding to periods of intensive sensor data collection and processing. The average load remains consistently below peak usage, indicating sufficient network capacity for controlling data transmission points. The system demonstrates NBU efficiency during off-peak (16–24 h), with data transfer rates stabilizing between 1.93 Mbps and 2.34 Mbps. The system’s efficient NBU during variable operational demands, robust network capacity planning, and efficient data transmission protocols are validated by its ability to handle intensive computational loads and maintain stable network operations across different scenarios, aligning with typical plant operation cycles.

## Conclusion and future work

This study proposes a novel alternative to traditional WWTP effluent quality prediction and control systems by introducing an ensemble DL model that integrates CNN + LSTM + GRU as base learners, combined through a QR-RF meta-learner. The predictive model was implemented within the AQUAtec-ICS and validated using large-scale operational data collected from the Jiangsu Municipal WWTCS, China. The proposed model achieved an MAE of 0.85 mg/L for short-term (1-h) COD concentration predictions, outperforming benchmark models, including Deep-LSTM and XGBoost-ARIMA, by 24% and 31% margins. The system demonstrated robust predictive performance across various operational conditions, achieving a PICP of 96.2% and maintaining high COD removal efficiency at 94.23%, compared to 88.76% achieved by conventional systems. Furthermore, operational deployment simulations indicated a 17% reduction in EC and a 24% optimization in chemical usage compared to standard WWTP control methodologies. The computational scalability analysis confirmed that the proposed system could efficiently manage up to 5000 *RPM*, maintaining stable CPU, GPU, and memory utilization under peak processing loads. These concrete achievements validate the scientific contribution of the proposed ensemble DL and ICS integration model, combining predictive accuracy, uncertainty quantification, and real-time operational decision-making to enhance WWTP efficiency and sustainability. Future research will focus on enhancing the model’s resilience under extreme environmental conditions such as sudden hydraulic or pollutant load shocks, integrating advanced meta-learning approaches for real-time hyperparameter tuning, and extending the predictive-control model to multi-plant systems operating under distributed control models.

## Data Availability

The datasets used and/or analyzed during the current study are available from the corresponding author upon reasonable request.
